# Stereotactic body radiation therapy and thermal ablation for treatment of patients with pulmonary metastases: a systematic literature review and meta-analysis

**DOI:** 10.1186/s12890-025-03561-9

**Published:** 2025-04-23

**Authors:** Paul Laeseke, Calvin Ng, Nicole Ferko, Andrada Naghi, George W.J. Wright, Di Wang, Alyshia Laidlaw, Iftekhar Kalsekar, Tony Amos, Balaji Laxmanan, Sudip K. Ghosh, Meijia Zhou, Philippe Szapary, Michael Pritchett

**Affiliations:** 1 Radiology, University of Wisconsin, 600 Highland Avenue, Madison, WI 53792 USA; 2 Department of Surgery, The Chinese University of Hong Kong, Prince of Wales Hospital, Shatin, NT, Hong Kong China; 3 EVERSANA, Burlington, ON Canada; 4 Lung Cancer Initiative, Johnson & Johnson, New Brunswick, NJ USA; 5 Health Economics and Market Access, Johnson & Johnson, Cincinnati, OH USA; 6 Medical Technology Epidemiology and Real World Data Science, Johnson & Johnson, New Brunswick, NJ USA; 7 Pulmonary and Critical Care Medicine, FirstHealth Moore Regional Hospital, and Pinehurst Medical Clinic, Pinehurst, NC USA

**Keywords:** Systematic literature review, Stereotactic body radiation therapy, Microwave ablation, Radiofrequency ablation, Meta regression, Meta-analysis, Oligometastases, Radioresistant

## Abstract

**Objective:**

To compare local tumor progression (LTP) and overall survival (OS) after image-guided thermal ablation (IGTA; microwave/radiofrequency ablation) versus stereotactic body radiation therapy (SBRT) in patients with pulmonary metastases.

**Methods:**

A systematic literature review was performed to capture studies that used IGTA or SBRT for patients with pulmonary metastases and studies that reported one, two, and threeyear LTP/OS were included. Patients with pulmonary metastases, and a subgroup with metastases from colorectal or renal cell carcinoma, or sarcoma (termed subgroup) which are considered more radioresistant, were analyzed. Single-arm pooled analyses, univariable, and multivariable random-effects meta-regressions were conducted to compare LTP and OS between IGTA and SBRT treated patients.

**Results:**

Analyses included 3,264 IGTA and 5,486 SBRT patients. IGTA patients with pulmonary metastases had higher LTP than SBRT patients at one year, 13% and 9% respectively. At two years, the LTP for IGTA patients was 14% compared to 16% for SBRT patients. Three-year LTP remained lower for IGTA patients compared to SBRT patients (14% and 22% respectively). In the subgroup, SBRT patients had higher LTP than IGTA patients across all timepoints. OS was similar across analyses/subgroups in the single-arm pooled analyses. The multivariable analyses showed that SBRT was associated with significantly lower OS at one year; however nonsignificant differences were observed at years two and three.

**Conclusions:**

In patients with pulmonary metastases, IGTA had lower LTP than SBRT at later timepoints. In patients with colorectal, renal cell carcinoma, or sarcoma pulmonary metastases, LTP was similar to overall LTP for IGTA, while it was higher for SBRT.

**Supplementary Information:**

The online version contains supplementary material available at 10.1186/s12890-025-03561-9.

## Background

Twenty-five to thirty percent of patients with malignant tumors will eventually get a pulmonary metastasis, making it one of the most frequent sites of tumor metastasis [1, 2]. Common primary tumors that metastasize to the lung include the breast, lung, colorectal, head/neck, kidney, skin, gynecological organs, and sarcomas [3, 4]. Survival of patients with pulmonary metastases remains inadequate with five-year survival rates ranging from 29% to 94% (dependent upon primary tumor histology) [3]. Systemic chemotherapy is a hallmark of treatment in this patient population, however, these patients are still considered incurable [5].

Local treatments can be an option for a subset of patients with limited metastatic disease. Surgery (referred to as metastasectomy) can be an effective treatment, however, some patients may not be eligible due to presence of multiple tumors, tumor location, comorbidities, or inadequate pulmonary reserve [6, 7]. Other non-surgical local treatments such as image-guided thermal ablation (IGTA) (radiofrequency ablation [RFA] and microwave ablation [MWA]) or stereotactic body radiation therapy (SBRT) are also used in clinical practice [5]. Microwave ablation is performed by inserting an antenna into tumors, heating the tissue to approximately 100°C with application of an electromagnetic field that causes dielectric polarization, resulting in coagulation of neoplastic cells and parenchyma, leading to necrosis [8–15]. For RFA, frequencies from 450 to 500 kHz are applied resulting in resistive heating in the tissue surrounding the electrode [12, 16]. Stereotactic body radiation therapy is an external beam radiation therapy that delivers a high dose of radiation to damage cancer cells in fractionated doses [17].

Studies comparing non-surgical approaches such as IGTA and SBRT are lacking, making comprehensive assessment of these different techniques challenging. SBRT may have limited efficacy in lung tumors with relatively radioresistant histologies (e.g., colorectal carcinoma [CRC], renal cell carcinoma [RCC], and sarcoma) [18]. Thus, as tumor histology may be an important predictor of outcomes in patients with pulmonary metastases, exploration of the outcome differences between SBRT and IGTA in patients with lung tumors from these specific histologies may be valuable. Moreover, to date there are no systematic literature reviews (SLRs) and metaanalyses comparing IGTA to SBRT in the treatment of patients with pulmonary metastases. The aim of this study was to compare local tumor progression (LTP) and overall survival (OS) after IGTA and SBRT in patients with pulmonary metastases using single-arm pooled metaanalyses and metaregressions.

## Methods

The Preferred Reporting Items for Systematic Reviews and Meta-Analysis (PRISMA) 2009 guidelines [19] were followed for this report and did not require institutional review board approval. The protocol for this SLR was not prospectively registered but the review followed predefined screening and extraction criteria. The methods used here were similar to those from previously conducted meta-analyses [20, 21]. The evidence screened for this systematic literature review and meta-analysis was based on a larger review in lung cancer where NSCLC analyses were previously published [21].

### Search strategies

MEDLINE^®^, Embase^®^, Evidence Based Medicine Cochrane Central Register of Controlled Trials, and the Cochrane Database of Systematic Reviews were used to perform the systematic searches. The original search was conducted on November 2nd, 2018 with annual updates until January 16th, 2022 (see Tables 1, 2, 3 and 4 in the Online Supplement). A medical information specialist used controlled vocabulary/keywords (i.e., metasta*, ablation, SBRT, lung) to develop search strategies that included English studies published 2005 or later. Another senior information specialist peer reviewed the search strategies prior to completing the search using the Peer Review of Electronic Search Strategies checklist [22].

### Study selection

The PICOS (i.e., population, intervention/comparator, outcomes, study design) criteria for study selection were developed a priori. Studies that included adult patients with pulmonary metastases that were treated with IGTA or SBRT and assessed LTP and OS were included (see Table 5 in the Online Supplement). To expand the evidence base, comparative, single-arm, and data from comparative studies that included only one comparator of interest were included. After the title and abstract of each record was screened, eligible studies moved to full-text screening. Duplicate review of each record was performed; discrepancies between reviewers were resolved by consensus or through adjudication from a third reviewer.

### Data extraction and study quality assessment

Study, patient, and intervention characteristics, and outcome data were extracted from each study (reported in text or digitized from Kaplan–Meier curves using DigitizeIt 2.5.3, Braunschweig, Germany). Data that included patients from the same institution or database with overlapping date ranges were not extracted. Local tumor progression was defined as progression at or adjacent to the treatment site [20] and was analyzed per tumor and per patient. If studies did not report both per tumor and per patient data, conversion between LTP reported per patient and per tumor (or vice versa) was performed using the average number of tumors in the study or subgroup.

A modified version of the methodological index for non-randomized studies (MINORS) tool was used to evaluate study quality (see Table 6 of the Online Supplement) [23]. Studies were scored on the description of an aim, inclusion of consecutive patients, study design, clear definitions of LTP and OS, unbiased assessment of study endpoints, length of follow-up, loss to follow-up rate and prospective calculation of study size. Studies could receive a score from 0 to 2 for each question. Data were extracted by a single reviewer and checked for accuracy by another with discrepancies resolved by consensus, or through adjudication from a third reviewer. If a study scored 7 or more on the MINORS tool, it was deemed as highquality. Use of this definition excluded 30.7% of the evidence base in a sensitivity analysis of highquality studies. Study quality assessments were performed to evaluate the reliability of the meta-analyses and meta-regression results.

### Data synthesis and statistical methods

#### Single-arm pooled analyses and publication bias

Single-arm pooled analyses were completed with a random effects meta-analytic model by combining the study-specific outcome rates and calculating the 95% confidence intervals (CIs) with the inverse variance method [20]. Forest plots were created from the results of these analyses with the R (version 3.6.1, Vienna, Austria) meta package. The LFK index was used to assess publication bias (index range: -1 to + 1 indicates no publication bias) [24] and Egger’s test was used to confirm these results (*P* < 0.05 statistically significant). Funnel asymmetry was also assessed in funnel plots created with the R (version 3.6.1, Vienna, Austria) meta package.

#### Meta-regressions

Univariable and multivariable random-effects meta-regressions were conducted to adjust for heterogeneity between included studies and to investigate the potential effects of prognostic factors on outcomes [20]. Meta-regression is recognized as an important statistical method to explore heterogeneity between studies within meta-analysis as it provides pooled effect estimates after adjustment of included covariates [25, 26]. Moreover, the use of meta-regression allows the testing of several covariates simultaneously to understand the extent of heterogeneity among the included studies [27]. Studies were weighted by sample size to evaluate the association between the following studylevel covariates: treatment (SBRT versus IGTA), proportion male, age, average tumor number, average tumor size, study design (prospective versus retrospective), geographic region (North America, Europe, international, Australia, and Asia as the reference group), and imaging method for LTP assessment (computed tomography [CT] versus CT and/or fluorodeoxyglucose [FDG]-positron emission tomography [PET]/PETCT as the reference group) [20]. Studies were excluded from the multivariable meta-regressions if they did not report a covariate of interest. The odds ratios (ORs) and the 95% CI for the treatment covariate were plotted on a log scale. If the 95% CI of a covariate’s OR excluded the null value of 1, the covariate was considered statistically significant. Meta-regressions were conducted with the R (version 3.6.1, Vienna, Austria) meta package.

#### Subgroup analyses

A subgroup of patients with pulmonary metastases from primary CRC, RCC, or sarcoma were analyzed in single-arm pooled meta-analyses (termed subgroup). This subgroup was analyzed separately as outcome differences in patients with pulmonary metastases have been shown to be related to tumor histology [28].

## Results

### Literature search and study characteristics

**Fig. 1:**
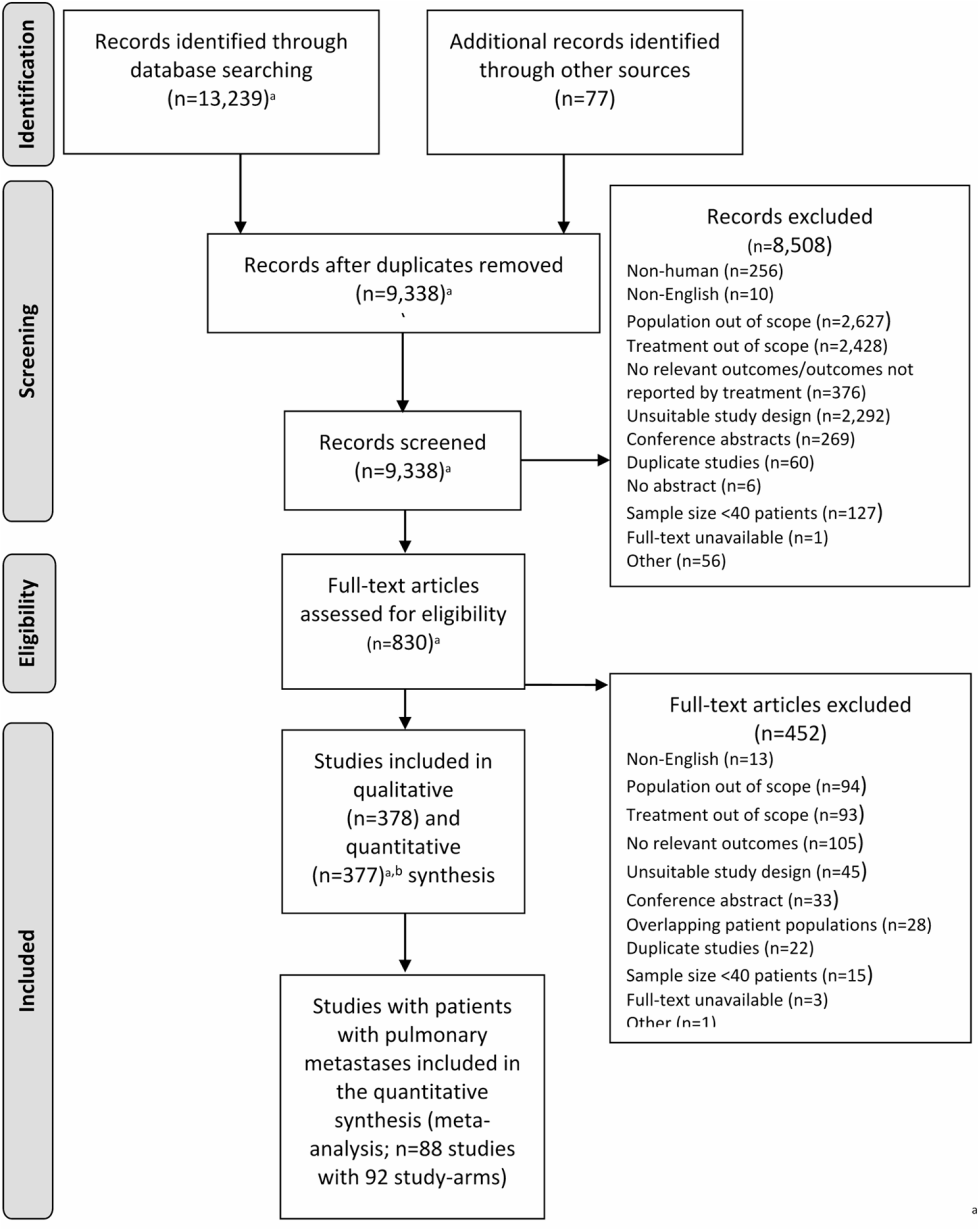
PRISMA flow diagram for studies with patients with pulmonary metastases Includes studies with NSCLC and patients with pulmonary metastases. See Sect. 9.0 of the Online Supplement for a list of included studies with patients with pulmonary metastases. ^b^ Two studies with duplicate patients combined for the quantitative synthesis. Abbreviations: NSCLC = non-small cell lung cancer; PRISMA = Preferred Reporting Items for Systematic Reviews

The systematic search for studies on MWA, RFA, and SBRT identified 13,316 records which included non-small cell lung cancer (NSCLC) and patients with pulmonary metastases. After the removal of duplicate records, 9,338 abstracts were screened with 8,508 excluded (e.g., nonhuman, population out of scope; Fig. 1). Eight hundred thirty studies that included NSCLC and patients with pulmonary metastases were screened in fulltext and 452 studies were excluded. Ninety two arms (from 88 studies) were included that enrolled patients with pulmonary metastases who were treated with IGTA (*n* = 35 study arms with 3,264 patients) or SBRT (*n* = 57 study arms with 5,486 patients; Table 1). Due to the PICOS criteria (i.e., inclusion of patients with pulmonary metastases and active sites of disease outside the lung), the patients with pulmonary metastases group analyzed here may include some stage IV NSCLC patients that were amenable to local treatment. However, the results of this manuscript will refer to this group as simply patients with pulmonary metastases, and additionally will refer to study arms as studies for brevity. Table 1 presents baseline characteristics for included studies on patients with pulmonary metastases. Table 7 of the Online Supplement provides the type of primary cancer for both study arms.

**Table 1:** Baseline characteristics for included studies

Continuous Variables	IGTA (MWA or RFA) (*N* = 35)		SBRT (*N* = 57)	
	Study Count	Mean (SD)	Study Count	Mean (SD)
Average age in years	24	63.5 (4.5)	40	67.8 (3.5)
Gender (% male)	26	55.44 (12.6)	44	59.7 (11.7)
Average tumor size (cm)	18	1.8 (0.8)	20	1.7 (0.3)
Average tumor number	29	1.9 (0.5)	48	1.6 (0.5)
Average % of patients with primary tumors from CRC, RCC or sarcoma	35	50.5 (41.3)	57	47.0 (33.5)
**Categorical Variables**	**Study Count**	**% of Total**	**Study Count**	**% of Total**
Design				
Retrospective	24	68.6%	49	86.0%
Prospective	11	31.4%	8	14.0%
Region				
Asia	13	37.1%	15	26.3%
North America	3	8.6%	7	12.3%
Europe	13	37.1%	28	49.1%
International	3	8.6%	2	3.5%
Australia	3	8.6%	5	8.8%

Abbreviations: CRC = colorectal carcinoma; IGTA = image-guided thermal ablation; MWA = microwave ablation; RCC = renal cell carcinoma; RFA = radiofrequency ablation; SBRT = stereotactic body radiation therapy; SD = standard deviation

### Local tumor progression analyzed per patient

**Fig. 2:**
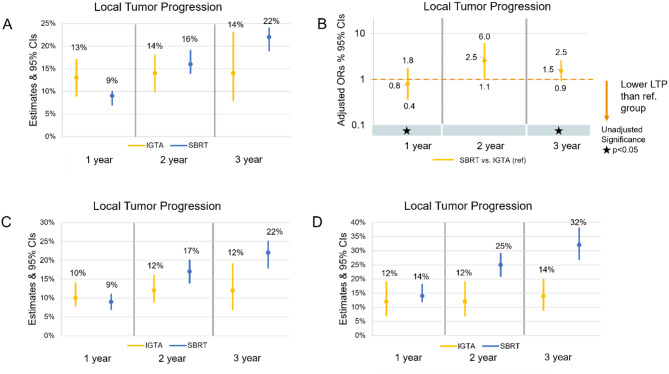
LTP analyzed per patient. **A**) point estimates from single-arm pooled analyses with 95% CIs, **B**) multivariable metaregressions, ^a^95% CIs, and univariable meta-regression significance, **C**) point estimates from single-arm pooled analyses with 95% CIs for studies with MINORS score of 7 or above and **D**) point estimates from single-arm pooled analyses with 95% CIs for patients with pulmonary metastases from CRC, RCC, or sarcoma. ^a^ Multivariable meta-regression adjusted for the following covariates: average age, % male, average tumor size and number, study design (retrospective versus prospective), geographic region (North America, Europe, international, Australia, and Asia as the reference group), and imaging method for LTP assessment (CT versus CT and/or FDG-PET/PETCT). Abbreviations: CI = confidence interval; CRC = colorectal carcinoma; CT = computed tomography; FDG = fluorodeoxyglucose; IGTA = image-guided thermal ablation; LTP = local tumor progression; ORs = odds ratios; PET = positron emission tomography; RCC = renal cell carcinoma; ref.=reference group; SBRT = stereotactic body radiation therapy

In point estimates from single-arm pooled analyses, LTP rates were lower (9% vs. 13%) for SBRT patients at one year, and lower for IGTA patients at two and three years (14% vs. 16%, and 14% vs. 22%, respectively) (Fig. 2A). This trend was similar in the univariable analyses, where patients treated with IGTA had higher LTP than SBRT patients at 1-year (*P* = 0.021) and lower LTP at 2 and 3 years (*P* = 0.403 and *P* = 0.031, respectively) (Fig. 2B). In multivariable metaregressions, SBRT treatment was associated with significantly higher LTP compared to IGTA at two years (Fig. 2B).

Studies with a MINORS score of 7 or greater were considered high-quality, and 70% of all studies had this designation (29 IGTA studies and 42 SBRT studies). In point estimates from single-arm pooled analyses for studies with MINORS scores of 7 or above, LTP followed a similar trend to that of the overall pulmonary metastases study group (Fig. 2C). However, the difference between 1-year LTP point estimates for SBRT and IGTA was smaller for the high-quality studies (9% and 13%, respectively for the overall group; 9% and 10%, respectively for high-quality studies). In the subgroup, patients treated with SBRT had higher LTP than patients treated with IGTA at all time points (Fig. 2D).

### Local tumor progression analyzed per tumor

**Fig. 3:**
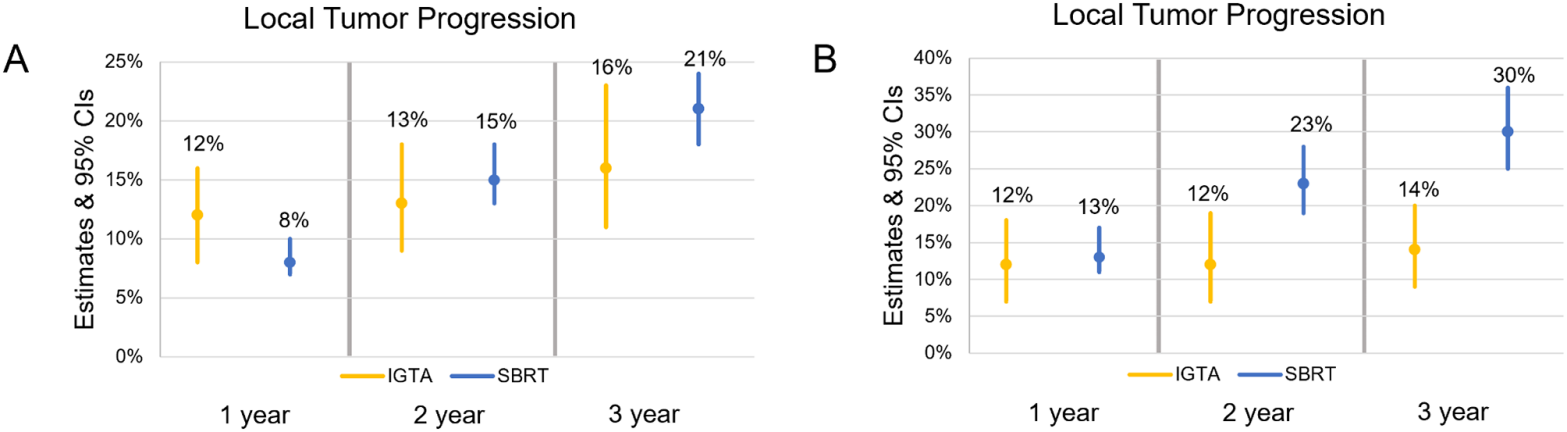
LTP analyzed per tumor. **A**) point estimates from single-arm pooled analyses with 95% CIs and **B**) point estimates from single-arm pooled analyses with 95% CIs for patients with pulmonary metastases from CRC, RCC, or sarcoma Abbreviations: CI = confidence interval; CRC = colorectal carcinoma; IGTA = image-guided thermal ablation; LTP = local tumor progression; RCC = renal cell carcinoma; SBRT = stereotactic body radiation therapy

For patients with pulmonary metastases analyzed per tumor, results were similar to per patient analysis results: LTP point estimates were lower for SBRT patients at one year (8% vs. 12%), and lower for IGTA patients at two and three years (13% vs. 15%, and 16% vs. 21%, respectively; Fig. 3A). Results were also similar to the overall analysis for the subgroup, where patients treated with SBRT had higher LTP than patients treated with IGTA at all time points (Fig. 3B).

### Overall survival

**Fig. 4:**
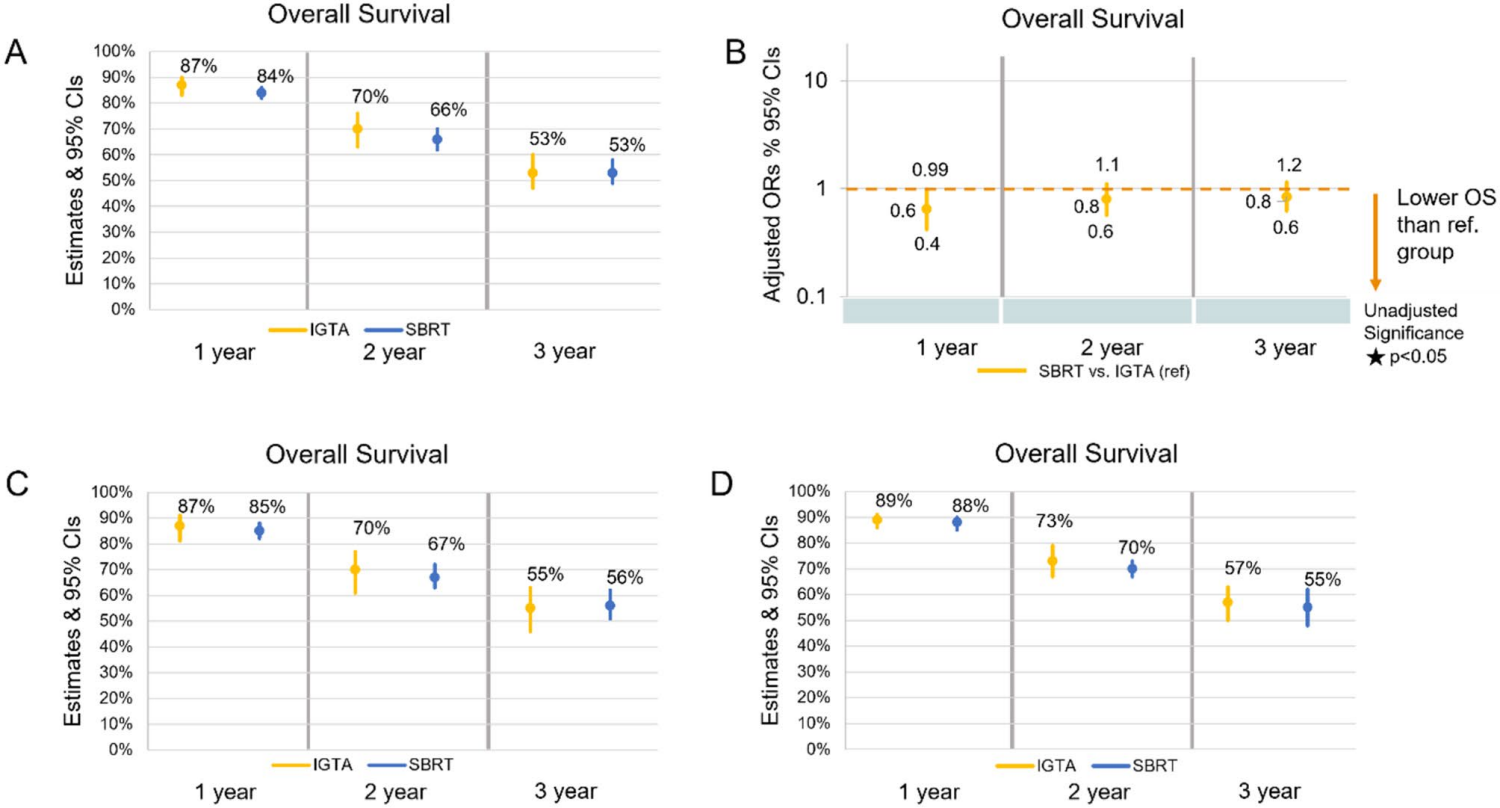
Overall survival by patient. **A**) point estimates from single-arm pooled analyses with 95% CIs, **B**) multivariable metaregressions,^a^ 95% CIs, and univariable meta-regression significance, **C**) point estimates from single-arm pooled analyses with 95% CIs for studies with MINORS score of 7 or above and **D**) point estimates from single-arm pooled analyses with 95% CIs for patients with pulmonary metastases from CRC, RCC, or sarcoma. ^a^ Multivariable meta-regression adjusted for the following covariates: average age, % male, average tumor size and number, study design (retrospective versus prospective), geographic region (North America, Europe, international, Australia, and Asia as the reference group), and imaging method for LTP assessment (CT versus CT and/or FDG-PET/PETCT). Abbreviations: CI = confidence interval; CRC = colorectal carcinoma; CT = computed tomography; FDG = fluorodeoxyglucose; IGTA = image-guided thermal ablation; ORs = odds ratios; OS = overall survival; ref. = reference group; PET = positron emission tomography; RCC = renal cell carcinoma; SBRT = stereotactic body radiation therapy

In single-arm pooled analyses, overall survival rates were higher for IGTA versus SBRT at one and two years (87% vs. 84% and 70% vs. 66%, respectively), and equal for both at 3 years (53%) (Fig. 4A). A similar trend was observed in univariable metaregressions, where OS was higher after IGTA for all time points, although the difference was not significant at any time point (Fig. 4B). In multivariable analyses, patients treated with SBRT had significantly lower OS than those treated with IGTA at one year; however, there were no significant differences at later time points (Fig. 4B).

In the sensitivity analysis of high-quality studies, OS followed a similar trend to that of the overall pulmonary metastases study group (Fig. 4C), except at the 3-year timepoint where the point estimate was 1% lower for IGTA compared to SBRT. In the subgroup, OS was higher after treatment with IGTA at all time points; differences between the point estimates for IGTA and SBRT were comparable to those of the overall pulmonary metastases group (Fig. 4D).

### Publication bias

The LFK index indicated that there was evidence of publication bias in all SBRT and IGTA analyses except 1-year LTP and 2-year OS for IGTA studies towards reporting more favorable outcomes for each treatment considered separately. These results were generally confirmed with the Egger’s test, although it was less sensitive at detecting possible publication bias in the IGTA group (see **Sect. 8.0** of the Online Supplement).

## Discussion

This systematic review used single-arm pooled analyses and meta-regressions to compare IGTA to SBRT in patients with pulmonary metastases from any primary origin and with those from primary CRC, RCC, or sarcoma. Analyses demonstrated that patients with pulmonary metastases treated with IGTA had higher OS at one year and lower LTP at two years compared to those treated with SBRT. Notably, this trend remained consistent when a subgroup of studies deemed as high quality were evaluated and when LTP was analyzed as per patient or per tumor. For LTP, SBRT rates were higher in the subgroup than the overall analysis, while this differential effect was not observed for IGTA.

There is some evidence indicating that local control may provide a survival benefit to oligometastatic patients. The evidence is most robust for surgical metastasectomy, as highlighted by the 1997 International Registry of Lung Metastases in a group of 5,206 patients undergoing pulmonary resection for oligometastases with primary tumors of mixed histology [29]. Patients who underwent complete resection experienced a clear OS advantage versus those who underwent incomplete resection (5-year OS of 36% versus 13%, respectively). The value of local control by metastasectomy is further supported by more recent observational studies in single-histology oligometastases to the lung patient populations: complete resection (vs. incomplete resection) in CRC and RCC patients, was found to be independently associated with improved survival [30–32]. Newer local treatment options, such as IGTA and SBRT, show a similar trend, however, there are fewer studies in this space and they have a smaller sample size [33]. In a study of 50 CRC patients with metastases to the lung treated with MWA, LTPfree survival (LTPFS) was significantly associated with an ablation margin size of < 5 mm (*P* < 0.001; direction not reported); LTP was only observed for tumors ablated with margins of < 5 mm (LTP rate of 24% in these tumors [6]. Lastly, for SBRT, the SABR-COMET trial (randomized 99 patients 1:2 to standard of care [SOC] or SOC + SBRT) highlighted a similar trend, with lower LTP and higher OS after treatment with SBRT (local control: 63% [37% LTP] at median-follow-up [MFU] of 51 months, and 5-year OS: 42.3%) compared to the control group (local control: 46% [54% LTP] at MFU, and 5-year OS: 17.7%) [34].

In this analysis, the entire patient cohort received treatment for pulmonary metastases, and there were no differences in LTP between IGTA and SBRT at one year. This may be because, at earlier time points, SBRT can cause radiationinduced pulmonary parenchymal changes and fibrosis, and CT scans can have difficulty distinguishing between these changes or local recurrence [35, 36]. At one year, 31% of SBRT studies used CT to assess LTP, while 69% of these studies used CT and/or FDG-PET/PET-CT. SBRT studies using CT alone for post assessment may underestimate progression due to radiation induced fibrosis and scarring. However, at two years, IGTA patients had lower LTP compared to SBRT patients (*P* = 0.037), with a similar trend seen at three years (*P* = 0.088). To understand the reason for this difference, the impact of primary tumor type on two- and three-year LTP was evaluated. At these time points, there were approximately 30% more IGTA than SBRT patients that had pulmonary metastases from primary CRC, RCC, or sarcoma. Since CRC, RCC, and sarcoma are considered more radioresistant compared to other tumor types [18, 28], IGTA may have better efficacy than SBRT in these patients. To control for this effect, a sensitivity analysis was conducted that controlled for the proportion of patients within a study that had pulmonary metastases from primary CRC, RCC, or sarcoma (added as an additional covariate), and included an interaction term that evaluated the effects of this additional covariate on treatment (data not reported). These analyses showcased lower LTP at one year for SBRT compared to IGTA when no patients had pulmonary metastases from primary CRC, RCC, or sarcoma (OR = 0.077, 95% CI: 0.008–0.721). Additionally, these analyses supported the hypothesis that studies with higher proportions of patients with pulmonary metastases from primary CRC, RCC, or sarcoma were more likely to administer IGTA over SBRT at one and two years (OR = 23.45, 95% CI: 1.60–342.41; OR = 38.55, 95% CI: 1.50–995.26, respectively).

Multiple SLRs and meta-analyses have demonstrated similar results to the current analysis [37]. A study by Nguyenhuy et al., 2022 analyzed 23 ablation studies including MWA, RFA, and cryoablation, in patients with pulmonary metastases [38]. The Nguyenhuy et al. one-year LTP was 9% (based on 1-year local effectiveness of 91%), similar to the 1-year LTP from IGTA and SBRT from this analysis of 13% and 9%, respectively [38]. However, the Nguyenhuy et al. 1- and 3-year OS rates (95.6% and 76.4%, respectively) in a subgroup of oligometastatic patients or those treated with curative intent, were higher than the current analysis (87% and 53%, respectively). Unlike the current analysis, Nguyenhuy et al. only included studies that labelled patients as oligometastatic or treated with curative intent. Thus, it is more likely that Nguyenhuy et al. included an oligometastatic patient cohort, while the current analysis likely has some patients that are not oligometastatic, and thus may have a decreased life expectancy [39, 40]. For SBRT, a metaanalysis published in 2022 that included 13 studies with patients with pulmonary metastases reported 3-year local control (77%, 23% LTP) and 3-year OS (41%) that was similar to the current analysis (3-year LTP: 22%, 3-year OS: 53%) [41]. In the current analyses, there were minimal survival differences observed between patients treated with IGTA versus SBRT.

An SLR and meta-analysis by Cao et al. published in 2019 included 18 SBRT studies that reported outcomes for patients with pulmonary metastases from CRC primaries [42]. Patients with CRC primaries that metastasized to the lung had slightly higher 1-to-3-year LTP (19%, 34%, 40%, respectively) than the current analysis (14%, 25%, and 32%, respectively) [42]. Higher LTP in this patient subgroup was also demonstrated in Choi et al., 2020 [43]. The Choi et al. meta-analysis included 14 SBRT studies on patients with pulmonary metastases from CRC primaries [43]. Higher LTP for patients with pulmonary metastases from CRC primaries when compared to nonCRC primaries has been shown previously (hazard ratio, 2.93; 95% CI: 1.93–4.45; *P* < 0.00001) [42] and may explain the differences seen in LTP rates between the patient cohort in the literature and in the subgroup of the current analysis. Lastly, metaanalyses on SBRT patients with pulmonary metastases from radioresistant histologies [43] and ablation patients with pulmonary oligometastases from CRC, RCC, or sarcoma primaries [38] have shown similar survival rates compared to the current analysis. Therefore, the type of primary cancer may influence the choice between IGTA and SBRT therapy for patients with pulmonary oligometastases. Current guidelines may not provide a distinction in treatment modalities for oligometastases based on the type of primary cancer. Additional research that focuses on the differences in LTP between patients with various primary cancers are warranted to confirm findings reported here, which may influence future treatment guidelines.

These findings should be interpreted in the context of the following limitations. First, most studies included in this SLR were single arm; thus, differences in patient populations and measurement variations can confound the comparisons of technologies. The meta-regression adjusted for study-level covariates (such as age, average tumor size, and geographical region), however, patient-level variability could not be controlled for due to lack of data [44]. Table 1 highlights the average baseline characteristics between IGTA and SBRT and shows that IGTA studies have fewer males (54%), a higher proportion of patients with primary tumors from CRC, RCC, or sarcoma (39.9%), lower mean age (63.2 years), and higher average number of tumors (1.83 tumors) compared to SBRT studies (60% male, 34.2% of patients with primary tumors from CRC, RCC, or sarcoma, 67.8 years old, and 1.54 average tumors). Additional comparative data from propensity-score matched comparative observational studies are warranted to support the present findings. Secondly, differences between treatment arms were also seen in the publication bias analyses, which suggested studies are more likely to report favorable outcomes for each treatment when considered separately. Publication bias toward more promising results has been demonstrated previously. Findings presented here are consistent with clinical study publishing practices, where studies that have poor results are less frequently published compared to studies that have promising results [45]. Typically, publication bias is assessed in SLRs that compare treatments to identify any bias towards one treatment over another. However, the publication basis analyses presented here evaluated the absolute treatment effects for both IGTA and SBRT separately. Lastly, this SLR did not include studies with oligometastatic cancer patients with tumors outside the lung that did not report lung-specific LTP data. Because of this, several SBRT studies were excluded from the current analysis. Additionally, other factors that characterize oligometastatic patients, such as timing of metastases (i.e., synchronous/metachronous), status of primary disease, and previous/concomitant therapies were not documented in most papers and hence were not part of the inclusion criteria in the current analysis.

Local tumor progression and OS in patients with pulmonary metastases treated with SBRT and IGTA were the focus of this meta-analysis. For implementation of these treatments in clinical practice, several other criteria such as safety profile, impact on patient’s health-related quality of life, convenience, and costs need to be considered. SBRT is commonly used to treat patients with pulmonary metastases; however, SBRT can cause complications, requires multiple treatment sessions, and can decrease pulmonary reserve [46]. Image-guided thermal ablation is an alternative treatment option for the management of patients with pulmonary metastases. Image-guided thermal ablation is advantageous to SBRT because it can be delivered in a single session, has lower procedural costs, and can be repeated [47]. While IGTA does not come with the same set of limitations as SBRT, percutaneous ablation is associated with an increased pneumothorax rate [48, 49]. Nevertheless, more data to confirm the results of this analysis and to explore complications, cost, and quality of life are needed. However, current data suggests that IGTA may be a viable option in the treatment of patients with pulmonary metastases.

## Conclusion

In conclusion, the results showed that patients treated with IGTA have similar LTP to SBRT at early time points and lower LTP at later time points. In the future, larger highquality comparative studies should be designed to confirm the findings of this analysis and to assess outcomes such as complications, resource use, and costs.

## Electronic supplementary material

Below is the link to the electronic supplementary material.

Supplementary Material 1: Tables 1, 2, 3 and 4: detailed search strategies, Table 5: PICOS criteria, Table 6: MINORS criteria, Table 7: primary tumor locations, Table 8: univariable analysis for LTP, and 9: univariable analyses for OS, Table 10: MINORS score per criterion for each study, Table 11: publication bias, Table 12: List of Included Studies, Fig. 1: study-level covariates associated with LTP or OS, Figs. 2 and 3 LTP and OS plots for publication bias.

## Data Availability

The data sets used and/or analyzed during the current study are available from the corresponding author on reasonable request.

## Abbreviations

CI: Confidence Interval
CRC: Colorectal carcinoma
CT: Computed tomography
FDG: Fluorodeoxyglucose
IGTA: Image-guided thermal ablation
LTP: Local tumor progression
LTPFS: Local tumor progression-free survival
MFU: Median follow-up
MINORS: Methodological index for non-randomized studies
MWA: Microwave ablation
NSCLC: Non-small cell lung cancer
OR: Odds ratio
OS: Overall survival
PET: Positron emission tomography
PICOS: Population, intervention, comparators, outcomes, study design
PRISMA: Preferred Reporting Items for Systematic Reviews and Meta-Analyses
RCC: Renal cell carcinoma
RFA: Radiofrequency ablation
SABR: Stereotactic ablative body radiotherapy
SBRT: Stereotactic body radiation therapy
SD: Standard deviation
SLR: Systematic literature review
SOC: Standard of care
