# Supplementary material for: Stereotactic body radiation therapy and thermal ablation for treatment of patients with pulmonary metastases: a systematic literature review and meta-analysis
Source: BMC Pulm Med. 2025 Apr 23;25:188. doi: 10.1186/s12890-025-03561-9 (PMC12016196; doi:10.1186/s12890-025-03561-9)
Supplement: Supplementary file 1 — Supplementary Material 1: Tables 1, 2, 3 and 4: detailed search strategies, Table 5: PICOS criteria, Table 6: MINORS criteria, Table 7: primary tumor locations, Table 8: univariable analysis for LTP, and 9: univariable analyses for OS, Table 10: MINORS score per criterion for each study, Table 11: publication bias, Table 12: List of Included Studies, Fig. 1: study-level covariates associated with LTP or OS, Figs. 2 and 3 LTP and OS plots for publication bias. [file 12890_2025_3561_MOESM1_ESM.docx]

**Supplementary Materials**

Table of Contents

[1.0 Search Strategies 2](#_Toc162900464)

[2.0 Detailed PICOS and Additional Data Extraction Information 19](#_Toc162900465)

[3.0 Quality Assessment of Studies (MINORS) 20](#_Toc162900466)

[4.0 Proportion of Primary Tumor Locations 23](#_Toc162900467)

[5.0 Patients with Pulmonary Metastases Meta-Regressions 24](#_Toc162900468)

[5.1 Patients with Pulmonary Metastases Tables for Univariable Analyses for LTP and OS 24](#_Toc162900469)

[6.0 Heat-Maps for Study-Level Covariates That Are Associated with LTP and OS 25](#_Toc162900470)

[7.0 MINORS Assessment of Studies 26](#_Toc162900471)

[8.0 Publication Bias 30](#_Toc162900472)

[9.0 Included Studies 33](#_Toc162900473)

# Search Strategies

Table 1: Database(s): Ovid MEDLINE(R) and Epub Ahead of Print, In-Process & Other Non-Indexed Citations and Daily 1946 to November 2nd, 2018 Search StrategyDatabase(s): Ovid MEDLINE(R) and Epub Ahead of Print, In-Process & Other Non-Indexed Citations and Daily 1946 to November 2^nd^, 2018 
Search Strategy:

| **#** | **Searches** | **Results** |
| --- | --- | --- |
| 1 | exp Lung Neoplasms/ | 213401 |
| 2 | ((neoplas* or cancer* or tumour* or tumor* or carcinoma* or malignan* or metasta* or oncolog*) adj3 (lung* or pulmonary or pleuropulmonary or pleural* or bronch*)).tw,kf. | 236551 |
| 3 | ((adenoma? or adenocarcinoma? or adeno-carcinoma? or blastoma? or carcinosarcoma? or carcino-sarcoma? or leukemia? or leukaemia? or lymphoma? or melanoma? or mesenchymoma? or mesothelioma? or sarcoma? or thymoma?) adj3 (lung* or pulmonary or pleuropulmonary or pleural* or bronch*)).tw,kf. | 33380 |
| 4 | (Pancoast adj (tumor* or tumour* or syndrome*)).tw,kf. | 402 |
| 5 | (SCLC or NSCLC).tw,kf. | 40976 |
| 6 | or/1-5 [LUNG CANCER] | 302805 |
| 7 | (microwave* adj3 (therap* or treatment*)).tw,kf. | 1729 |
| 8 | (microwave adj ablation*).tw,kf. | 1302 |
| 9 | MW ablation*.tw,kf. | 101 |
| 10 | MW coagulation*.tw,kf. | 1 |
| 11 | (MW adj (electrocoagulation* or electro-coagulation*)).tw,kf. | 0 |
| 12 | (MW adj (thermocoagulation* or thermo-coagulation*)).tw,kf. | 0 |
| 13 | (MW adj (electrocauter* or electro-cauter*)).tw,kf. | 0 |
| 14 | (MW adj (thermal therap* or thermotherap* or thermo-therap*)).tw,kf. | 4 |
| 15 | MWA.tw,kf. | 647 |
| 16 | (PMCT or PMCTs).tw,kf. | 349 |
| 17 | (PMAT or PMATs).tw,kf. | 97 |
| 18 | (RALTCT or RALTCTs).tw,kf. | 1 |
| 19 | (MCT or MCTs).tw,kf. | 5785 |
| 20 | or/7-19 [MICROWAVE ABLATION] | 9190 |
| 21 | Microwaves/ | 15878 |
| 22 | microwave*.tw,kf. | 32169 |
| 23 | (extremely high frequency adj (radiowave* or radio wave* or wave*)).tw,kf. | 0 |
| 24 | (EHF adj (radiowave* or radio wave* or wave*)).tw,kf. | 6 |
| 25 | ((ultrahigh or ultra-high) adj frequency adj (radiowave* or radio wave* or wave*)).tw,kf. | 15 |
| 26 | or/21-25 [MICROWAVES] | 34773 |
| 27 | (Acculis* or Amica* or AMICA-GEM* or Avecure* or Certus* or Emblation* or Emprint* or FORSEA* or MicrothermX* or MSYS245 or NEUWAVE* or Solero* or TATO or TATOPro*).tw,kf. | 490 |
| 28 | (Evident* and Covidien).tw,kf. | 6 |
| 29 | 27 or 28 [MICROWAVE ABLATION DEVICES/SYSTEMS] | 496 |
| 30 | ((radiofrequency or radio-frequency) adj3 (therap* or treatment*)).tw,kf. | 2638 |
| 31 | ((radiofrequency or radio-frequency) adj ablation*).tw,kf. | 13946 |
| 32 | RF ablation*.tw,kf. | 2294 |
| 33 | RF coagulation*.tw,kf. | 22 |
| 34 | (RF adj (electrocoagulation* or electro-coagulation*)).tw,kf. | 4 |
| 35 | (RF adj (thermocoagulation* or thermo-coagulation*)).tw,kf. | 20 |
| 36 | (RF adj (electrocauter* or electro-cauter*)).tw,kf. | 5 |
| 37 | (RF adj (thermal therap* or thermotherap* or thermo-therap*)).tw,kf. | 21 |
| 38 | RFA.tw,kf. | 5789 |
| 39 | PRFA.tw,kf. | 462 |
| 40 | EUS-RFA.tw,kf. | 14 |
| 41 | or/30-40 [RADIOFREQUENCY ABLATION] | 17875 |
| 42 | Radio waves/ | 7176 |
| 43 | Radiofrequency.tw,kf. | 30468 |
| 44 | (high frequency adj (current* or radiowave* or radio wave* or wave*)).tw,kf. | 550 |
| 45 | (HF adj (current* or radiowave* or radio wave* or wave*)).tw,kf. | 74 |
| 46 | Hertzian Wave*.tw,kf. | 9 |
| 47 | Short Wave*.tw,kf. | 4779 |
| 48 | or/42-47 [RADIO WAVES] | 39830 |
| 49 | ("RF 3000" or Cool Tip or StarBurst or RITA or Acculis or Uniblate or Habib).tw,kf. | 1826 |
| 50 | (Boston Scientific or Angiodynamics or Medtronic).tw,kf. | 5630 |
| 51 | or/49-50 [RF ABLATION DEVICES/SYSTEMS] | 7444 |
| 52 | exp Ablation Techniques/ | 106855 |
| 53 | ablat*.tw,kf. | 99459 |
| 54 | thermal heat*.tw,kf. | 427 |
| 55 | (thermal therap* or thermotherap* or thermo-therap*).tw,kf. | 3539 |
| 56 | Electrocoagulation/ | 11190 |
| 57 | coagulation*.tw,kf. | 92460 |
| 58 | (electrocoagulation* or electro-coagulation*).tw,kf. | 3519 |
| 59 | (thermocoagulation* or thermo-coagulation*).tw,kf. | 964 |
| 60 | (electrocauter* or electro-cauter*).tw,kf. | 3419 |
| 61 | or/52-60 [ABLATION/COAGULATION] | 264941 |
| 62 | 26 and 61 [MICROWAVES + ABLATION/COAGULATION] | 3329 |
| 63 | 48 and 61 [RADIO WAVES + ABLATION/COAGULATION] | 22087 |
| 64 | 20 or 29 or 41 or 51 or 62 or 63 [MICROWAVE/RADIOFREQUENCY ABLATION] | 42268 |
| 65 | 6 and 64 [LUNG CANCER AND ABLATION] | 1228 |
| 66 | exp Animals/ not (exp Animals/ and Humans/) | 4510754 |
| 67 | 65 not 66 [ANIMAL-ONLY REMOVED] | 1162 |
| 68 | exp Child/ not (exp Adult/ or Adolescent/) | 770725 |
| 69 | exp Infant/ not (exp Adult/ or Adolescent/) | 670380 |
| 70 | 67 not (68 or 69) [CHILD- AND INFANT-ONLY REMOVED] | 1156 |
| 71 | (comment or editorial or interview or news or newspaper article).pt. | 1292047 |
| 72 | (letter not (letter and randomized controlled trial)).pt. | 1000075 |
| 73 | 70 not (71 or 72) [OPINION PIECES REMOVED] | 1106 |
| 74 | randomized controlled trial.pt. | 470767 |
| 75 | controlled clinical trial.pt. | 92734 |
| 76 | randomized.ab. | 425567 |
| 77 | placebo.ab. | 192922 |
| 78 | randomly.ab. | 299729 |
| 79 | clinical trials as topic.sh. | 185171 |
| 80 | trial.ti. | 189478 |
| 81 | controlled clinical trial.pt. | 92734 |
| 82 | or/74-81 [RCTs] | 1180055 |
| 83 | Controlled Clinical Trial/ or Controlled Clinical Trials as Topic/ | 98063 |
| 84 | (control* adj2 trial*).tw,kf. | 233428 |
| 85 | Non-Randomized Controlled Trials as Topic/ | 415 |
| 86 | (nonrandom* or non-random* or quasi-random* or quasi-experiment*).tw,kf. | 49224 |
| 87 | (nRCT or nRCTs or non-RCT?).tw,kf. | 696 |
| 88 | Controlled Before-After Studies/ | 359 |
| 89 | (control* adj3 ("before and after" or "before after")).tw,kf. | 3866 |
| 90 | Interrupted Time Series Analysis/ | 493 |
| 91 | time series.tw,kf. | 26066 |
| 92 | (pre- adj3 post-).tw,kf. | 69865 |
| 93 | (pretest adj3 posttest).tw,kf. | 4708 |
| 94 | Historically Controlled Study/ | 145 |
| 95 | (control* adj2 stud$3).tw,kf. | 210339 |
| 96 | Control Groups/ | 1597 |
| 97 | (control* adj2 group$1).tw,kf. | 447979 |
| 98 | trial.ti. | 189478 |
| 99 | or/81-97 [NON-RCTS] | 1757203 |
| 100 | exp Cohort Studies/ | 1793475 |
| 101 | cohort?.tw,kf. | 483658 |
| 102 | Retrospective Studies/ | 714827 |
| 103 | (longitudinal or prospective or retrospective).tw,kf. | 1101568 |
| 104 | ((followup or follow-up) adj (study or studies)).tw,kf. | 47847 |
| 105 | Observational study.pt. | 54048 |
| 106 | (observation$2 adj (study or studies)).tw,kf. | 87784 |
| 107 | ((population or population-based) adj (study or studies or analys#s)).tw,kf. | 15713 |
| 108 | ((multidimensional or multi-dimensional) adj (study or studies)).tw,kf. | 103 |
| 109 | Comparative Study.pt. | 1813063 |
| 110 | ((comparative or comparison) adj (study or studies)).tw,kf. | 105812 |
| 111 | exp Case-Control Studies/ | 950949 |
| 112 | ((case-control* or case-based or case-comparison) adj (study or studies)).tw,kf. | 95227 |
| 113 | or/100-112 [OBSERVATIONAL STUDIES] | 4123412 |
| 114 | review.pt. | 2446203 |
| 115 | meta-analysis.pt. | 93800 |
| 116 | meta-analysis/ or systematic review/ or meta-analysis as topic/ or "meta analysis (topic)"/ or "systematic review (topic)"/ or exp technology assessment, biomedical/ | 119638 |
| 117 | ((systematic* adj3 (review* or overview*)) or (methodologic* adj3 (review* or overview*))).ti,ab,kf,kw. | 145442 |
| 118 | ((quantitative adj3 (review* or overview* or synthes*)) or (research adj3 (integrati* or overview*))).ti,ab,kf,kw. | 9187 |
| 119 | ((integrative adj3 (review* or overview*)) or (collaborative adj3 (review* or overview*)) or (pool* adj3 analy*)).ti,ab,kf,kw. | 21489 |
| 120 | (data synthes* or data extraction* or data abstraction*).ti,ab,kf,kw. | 22029 |
| 121 | (handsearch* or hand search*).ti,ab,kf,kw. | 8217 |
| 122 | (mantel haenszel or peto or der simonian or dersimonian or fixed effect* or latin square*).ti,ab,kf,kw. | 22573 |
| 123 | (met analy* or metanaly* or technology assessment* or HTA or HTAs or technology overview* or technology appraisal*).ti,ab,kf,kw. | 8134 |
| 124 | (meta regression* or metaregression*).ti,ab,kf,kw. | 6717 |
| 125 | (meta-analy* or metaanaly* or systematic review* or biomedical technology assessment* or bio-medical technology assessment*).mp,hw. | 237913 |
| 126 | (medline or cochrane or pubmed or medlars or embase or cinahl).ti,ab,hw. | 176695 |
| 127 | (cochrane or (health adj2 technology assessment) or evidence report).jw. | 18222 |
| 128 | (meta-analysis or systematic review).md. | 0 |
| 129 | (comparative adj3 (efficacy or effectiveness)).ti,ab,kf,kw. | 11576 |
| 130 | (outcomes research or relative effectiveness).ti,ab,kf,kw. | 8160 |
| 131 | ((indirect or indirect treatment or mixed-treatment) adj comparison*).ti,ab,kf,kw. | 1817 |
| 132 | or/114-131 [REVIEWS] | 2629630 |
| 133 | 73 and (82 or 99 or 113 or 132) | 632 |
| 134 | limit 133 to english language | 552 |
| 135 | limit 134 to yr="2005 -Current" | 496 |
| 136 | remove duplicates from 135 | 496 |

Table 2: Database(s): Embase 1974 to 2018 November 02 Search Strategy
Search Strategy:

| **#** | **Searches** | **Results** |
| --- | --- | --- |
| 1 | exp lung cancer/ | 269094 |
| 2 | ((neoplas* or cancer* or tumour* or tumor* or carcinoma* or malignan* or metasta* or oncolog*) adj3 (lung* or pulmonary or pleuropulmonary or pleural* or bronch*)).tw,kw. | 326677 |
| 3 | ((adenoma? or adenocarcinoma? or adeno-carcinoma? or blastoma? or carcinosarcoma? or carcino-sarcoma? or leukemia? or leukaemia? or lymphoma? or melanoma? or mesenchymoma? or mesothelioma? or sarcoma? or thymoma?) adj3 (lung* or pulmonary or pleuropulmonary or pleural* or bronch*)).tw,kw. | 48541 |
| 4 | (Pancoast adj (tumor* or tumour* or syndrome*)).tw,kw. | 506 |
| 5 | (SCLC or NSCLC).tw,kw. | 75043 |
| 6 | or/1-5 [LUNG CANCER] | 413680 |
| 7 | exp microwave thermotherapy/ | 1551 |
| 8 | (microwave* adj3 (therap* or treatment*)).tw,kw. | 2171 |
| 9 | (microwave adj ablation*).tw,kw. | 2183 |
| 10 | MW ablation*.tw,kw. | 196 |
| 11 | MW coagulation*.tw,kw. | 2 |
| 12 | (MW adj (electrocoagulation* or electro-coagulation*)).tw,kw. | 0 |
| 13 | (MW adj (thermocoagulation* or thermo-coagulation*)).tw,kw. | 0 |
| 14 | (MW adj (electrocauter* or electro-cauter*)).tw,kw. | 0 |
| 15 | (MW adj (thermal therap* or thermotherap* or thermo-therap*)).tw,kw. | 5 |
| 16 | MWA.tw,kw. | 1207 |
| 17 | (PMCT or PMCTs).tw,kw. | 536 |
| 18 | (PMAT or PMATs).tw,kw. | 171 |
| 19 | (RALTCT or RALTCTs).tw,kw. | 1 |
| 20 | (MCT or MCTs).tw,kw. | 8938 |
| 21 | or/7-20 [MICROWAVE ABLATION] | 14640 |
| 22 | microwave radiation/ | 20397 |
| 23 | microwave*.tw,kw. | 34790 |
| 24 | (extremely high frequency adj (radiowave* or radio wave* or wave*)).tw,kw. | 0 |
| 25 | (EHF adj (radiowave* or radio wave* or wave*)).tw,kw. | 5 |
| 26 | ((ultrahigh or ultra-high) adj frequency adj (radiowave* or radio wave* or wave*)).tw,kw. | 8 |
| 27 | or/22-26 [MICROWAVES] | 37928 |
| 28 | (Acculis* or Amica* or AMICA-GEM* or Avecure* or Certus* or Emblation* or Emprint* or FORSEA* or MicrothermX* or MSYS245 or NEUWAVE* or Solero* or TATO or TATOPro*).tw,kw. | 1169 |
| 29 | (Evident* and Covidien).tw,kw. | 35 |
| 30 | 28 or 29 [MICROWAVE ABLATION DEVICES/SYSTEMS] | 1203 |
| 31 | exp radiofrequency ablation/ | 28903 |
| 32 | ((radiofrequency or radio-frequency) adj3 (therap* or treatment*)).tw,kw. | 4142 |
| 33 | ((radiofrequency or radio-frequency) adj ablation*).tw,kw. | 23345 |
| 34 | RF ablation*.tw,kw. | 4363 |
| 35 | RF coagulation*.tw,kw. | 28 |
| 36 | (RF adj (electrocoagulation* or electro-coagulation*)).tw,kw. | 4 |
| 37 | (RF adj (thermocoagulation* or thermo-coagulation*)).tw,kw. | 32 |
| 38 | (RF adj (electrocauter* or electro-cauter*)).tw,kw. | 7 |
| 39 | (RF adj (thermal therap* or thermotherap* or thermo-therap*)).tw,kw. | 29 |
| 40 | RFA.tw,kw. | 11323 |
| 41 | PRFA.tw,kw. | 522 |
| 42 | EUS-RFA.tw,kw. | 42 |
| 43 | or/31-42 [RADIOFREQUENCY ABLATION] | 41354 |
| 44 | radiofrequency radiation/ | 5758 |
| 45 | Radiofrequency.tw,kw. | 46494 |
| 46 | (high frequency adj (current* or radiowave* or radio wave* or wave*)).tw,kw. | 528 |
| 47 | (HF adj (current* or radiowave* or radio wave* or wave*)).tw,kw. | 102 |
| 48 | Hertzian Wave*.tw,kw. | 7 |
| 49 | Short Wave*.tw,kw. | 4062 |
| 50 | or/44-49 [RADIO WAVES] | 54424 |
| 51 | ("RF 3000" or Cool Tip or StarBurst or RITA or Acculis or Uniblate or Habib).tw,kw. | 2636 |
| 52 | (Boston Scientific or Angiodynamics or Medtronic).tw,kw. | 13709 |
| 53 | or/51-52 [RF ABLATION DEVICES/SYSTEMS] | 16290 |
| 54 | exp ablation therapy/ | 41767 |
| 55 | ablat*.tw,kw. | 148271 |
| 56 | thermal heat*.tw,kw. | 474 |
| 57 | (thermal therap* or thermotherap* or thermo-therap*).tw,kw. | 4664 |
| 58 | exp electrocoagulation/ or thermocoagulation/ | 11965 |
| 59 | coagulation*.tw,kw. | 120613 |
| 60 | (electrocoagulation* or electro-coagulation*).tw,kw. | 3595 |
| 61 | (thermocoagulation* or thermo-coagulation*).tw,kw. | 1296 |
| 62 | (electrocauter* or electro-cauter*).tw,kw. | 5047 |
| 63 | or/54-62 [ABLATION/COAGULATION] | 291916 |
| 64 | 27 and 63 [MICROWAVES + ABLATION/COAGULATION] | 4644 |
| 65 | 50 and 63 [RADIO WAVES + ABLATION/COAGULATION] | 34687 |
| 66 | 21 or 30 or 43 or 53 or 64 or 65 [MICROWAVE/RADIOFREQUENCY ABLATION] | 80131 |
| 67 | 6 and 66 [LUNG CANCER AND ABLATION] | 2711 |
| 68 | exp animal experimentation/ or exp animal model/ or exp animal experiment/ or nonhuman/ or exp vertebrate/ | 24625174 |
| 69 | exp human/ or exp human experimentation/ or exp human experiment/ | 18989716 |
| 70 | 67 not (68 not 69) [ANIMAL-ONLY REMOVED] | 2622 |
| 71 | exp adolescent/ not (exp adult/ and exp adolescent/) | 512815 |
| 72 | exp child/ not (exp adult/ and exp child/) | 1740094 |
| 73 | fetus/ not (fetus/ and exp adult/) | 150421 |
| 74 | 70 not (71 or 72 or 73) [UNDER 18 REMOVED] | 2594 |
| 75 | editorial.pt. | 583811 |
| 76 | letter.pt. not (randomized controlled trial/ and letter.pt.) | 1036227 |
| 77 | 74 not (75 or 76) [OPINION PIECES REMOVED] | 2472 |
| 78 | conference abstract.pt. | 3206691 |
| 79 | 77 not 78 [CONFERENCE ABSTRACTS REMOVED] | 1829 |
| 80 | randomized controlled trial/ | 521459 |
| 81 | controlled clinical trial/ | 458408 |
| 82 | exp "clinical trial (topic)"/ | 279338 |
| 83 | (randomi#ed or randomi#ation? or randomly or RCT? or placebo*).tw,kw. | 1219971 |
| 84 | ((singl* or doubl* or trebl* or tripl*) adj (mask* or blind* or dumm*)).tw,kw. | 217689 |
| 85 | trial.ti. | 255269 |
| 86 | or/80-85 [RCTs] | 1732309 |
| 87 | exp controlled clinical trial/ | 703638 |
| 88 | exp "controlled clinical trial (topic)"/ | 158335 |
| 89 | (control* adj2 trial*).tw,kw. | 310822 |
| 90 | (nonrandom* or non-random* or quasi-random* or quasi-experiment*).tw,kw. | 61087 |
| 91 | (nRCT or nRCTs or non-RCT$1).tw,kw. | 1002 |
| 92 | (control* adj3 ("before and after" or "before after")).tw,kw. | 4991 |
| 93 | time series analysis/ | 21623 |
| 94 | time series.tw,kw. | 29482 |
| 95 | pretest posttest control group design/ | 354 |
| 96 | (pre- adj3 post-).tw,kw. | 123320 |
| 97 | (pretest adj3 posttest).tw,kw. | 5337 |
| 98 | controlled study/ | 6259128 |
| 99 | (control* adj2 stud$3).tw,kw. | 278591 |
| 100 | control group/ | 109603 |
| 101 | (control* adj2 group$1).tw,kw. | 634841 |
| 102 | trial.ti. | 255269 |
| 103 | or/87-102 [NON-RCTs] | 7095619 |
| 104 | cohort analysis/ | 415254 |
| 105 | cohort?.tw,kw. | 808958 |
| 106 | retrospective study/ | 705325 |
| 107 | longitudinal study/ | 118067 |
| 108 | prospective study/ | 481579 |
| 109 | (longitudinal or prospective or retrospective).tw,kw. | 1653980 |
| 110 | follow up/ | 1329005 |
| 111 | ((followup or follow-up) adj (study or studies)).tw,kw. | 62332 |
| 112 | observational study/ | 152563 |
| 113 | (observation$2 adj (study or studies)).tw,kw. | 136858 |
| 114 | population research/ | 94354 |
| 115 | ((population or population-based) adj (study or studies or analys#s)).tw,kw. | 22127 |
| 116 | ((multidimensional or multi-dimensional) adj (study or studies)).tw,kw. | 132 |
| 117 | exp comparative study/ | 1275031 |
| 118 | ((comparative or comparison) adj (study or studies)).tw,kw. | 119383 |
| 119 | exp case control study/ | 150406 |
| 120 | ((case-control* or case-based or case-comparison) adj (study or studies)).tw,kw. | 122204 |
| 121 | or/104-120 [OBSERVATIONAL STUDIES] | 4751891 |
| 122 | "review"/ | 2291129 |
| 123 | meta-analysis/ or systematic review/ or meta-analysis as topic/ or "meta analysis (topic)"/ or "systematic review (topic)"/ or exp technology assessment, biomedical/ | 321541 |
| 124 | ((systematic* adj3 (review* or overview*)) or (methodologic* adj3 (review* or overview*))).ti,ab,kw. | 181366 |
| 125 | ((quantitative adj3 (review* or overview* or synthes*)) or (research adj3 (integrati* or overview*))).ti,ab,kw. | 10761 |
| 126 | ((integrative adj3 (review* or overview*)) or (collaborative adj3 (review* or overview*)) or (pool* adj3 analy*)).ti,ab,kw. | 30634 |
| 127 | (data synthes* or data extraction* or data abstraction*).ti,ab,kw. | 27154 |
| 128 | (handsearch* or hand search*).ti,ab,kw. | 9937 |
| 129 | (mantel haenszel or peto or der simonian or dersimonian or fixed effect* or latin square*).ti,ab,kw. | 29135 |
| 130 | (meta regression* or metaregression*).ti,ab,kw. | 8406 |
| 131 | (meta-analy* or metaanaly* or systematic review* or biomedical technology assessment* or bio-medical technology assessment*).mp,hw. | 393333 |
| 132 | (medline or cochrane or pubmed or medlars or embase or cinahl).ti,ab,hw. | 234094 |
| 133 | (cochrane or (health adj2 technology assessment) or evidence report).jw. | 25394 |
| 134 | (comparative adj3 (efficacy or effectiveness)).ti,ab,kw. | 16590 |
| 135 | (outcomes research or relative effectiveness).ti,ab,kw. | 11914 |
| 136 | ((indirect or indirect treatment or mixed-treatment) adj comparison*).ti,ab,kw. | 3345 |
| 137 | or/122-136 [REVIEWS] | 2665677 |
| 138 | 79 and (86 or 103 or 121 or 137) | 1244 |
| 139 | limit 138 to english language | 1122 |
| 140 | limit 139 to yr="2005 -Current" | 1043 |
| 141 | remove duplicates from 140 | 1024 |

Table 3: Database(s): EBM Reviews - Cochrane Central Register of Controlled Trials September 2018, EBM Reviews - Cochrane Database of Systematic Reviews 2005 to October 31, 2018 Search Strategy

Search Strategy:

| **#** | **Searches** | **Results** |
| --- | --- | --- |
| 1 | exp Lung Neoplasms/ | 6432 |
| 2 | ((neoplas* or cancer* or tumour* or tumor* or carcinoma* or malignan* or metasta* or oncolog*) adj3 (lung* or pulmonary or pleuropulmonary or pleural* or bronch*)).tw,kw. | 15639 |
| 3 | ((adenoma? or adenocarcinoma? or adeno-carcinoma? or blastoma? or carcinosarcoma? or carcino-sarcoma? or leukemia? or leukaemia? or lymphoma? or melanoma? or mesenchymoma? or mesothelioma? or sarcoma? or thymoma?) adj3 (lung* or pulmonary or pleuropulmonary or pleural* or bronch*)).tw,kw. | 985 |
| 4 | (Pancoast adj (tumor* or tumour* or syndrome*)).tw,kw. | 4 |
| 5 | (SCLC or NSCLC).tw,kw. | 6878 |
| 6 | or/1-5 [LUNG CANCER] | 16961 |
| 7 | (microwave* adj3 (therap* or treatment*)).tw,kw. | 218 |
| 8 | (microwave adj ablation*).tw,kw. | 131 |
| 9 | MW ablation*.tw,kw. | 11 |
| 10 | MW coagulation*.tw,kw. | 0 |
| 11 | (MW adj (electrocoagulation* or electro-coagulation*)).tw,kw. | 0 |
| 12 | (MW adj (thermocoagulation* or thermo-coagulation*)).tw,kw. | 0 |
| 13 | (MW adj (electrocauter* or electro-cauter*)).tw,kw. | 0 |
| 14 | (MW adj (thermal therap* or thermotherap* or thermo-therap*)).tw,kw. | 0 |
| 15 | MWA.tw,kw. | 65 |
| 16 | (PMCT or PMCTs).tw,kw. | 13 |
| 17 | (PMAT or PMATs).tw,kw. | 3 |
| 18 | (RALTCT or RALTCTs).tw,kw. | 0 |
| 19 | (MCT or MCTs).tw,kw. | 809 |
| 20 | or/7-19 [MICROWAVE ABLATION] | 1141 |
| 21 | Microwaves/ | 201 |
| 22 | microwave*.tw,kw. | 610 |
| 23 | (extremely high frequency adj (radiowave* or radio wave* or wave*)).tw,kw. | 0 |
| 24 | (EHF adj (radiowave* or radio wave* or wave*)).tw,kw. | 1 |
| 25 | ((ultrahigh or ultra-high) adj frequency adj (radiowave* or radio wave* or wave*)).tw,kw. | 1 |
| 26 | or/21-25 [MICROWAVES] | 650 |
| 27 | (Acculis* or Amica* or AMICA-GEM* or Avecure* or Certus* or Emblation* or Emprint* or FORSEA* or MicrothermX* or MSYS245 or NEUWAVE* or Solero* or TATO or TATOPro*).tw,kw. | 48 |
| 28 | (Evident* and Covidien).tw,kw. | 8 |
| 29 | 27 or 28 [MICROWAVE ABLATION DEVICES/SYSTEMS] | 56 |
| 30 | ((radiofrequency or radio-frequency) adj3 (therap* or treatment*)).tw,kw. | 685 |
| 31 | ((radiofrequency or radio-frequency) adj ablation*).tw,kw. | 1355 |
| 32 | RF ablation*.tw,kw. | 241 |
| 33 | RF coagulation*.tw,kw. | 3 |
| 34 | (RF adj (electrocoagulation* or electro-coagulation*)).tw,kw. | 0 |
| 35 | (RF adj (thermocoagulation* or thermo-coagulation*)).tw,kw. | 2 |
| 36 | (RF adj (electrocauter* or electro-cauter*)).tw,kw. | 1 |
| 37 | (RF adj (thermal therap* or thermotherap* or thermo-therap*)).tw,kw. | 6 |
| 38 | RFA.tw,kw. | 715 |
| 39 | PRFA.tw,kw. | 17 |
| 40 | EUS-RFA.tw,kw. | 5 |
| 41 | or/30-40 [RADIOFREQUENCY ABLATION] | 2017 |
| 42 | Radio waves/ | 166 |
| 43 | Radiofrequency.tw,kw. | 3000 |
| 44 | (high frequency adj (current* or radiowave* or radio wave* or wave*)).tw,kw. | 25 |
| 45 | (HF adj (current* or radiowave* or radio wave* or wave*)).tw,kw. | 21 |
| 46 | Hertzian Wave*.tw,kw. | 0 |
| 47 | Short Wave*.tw,kw. | 315 |
| 48 | or/42-47 [RADIO WAVES] | 3403 |
| 49 | ("RF 3000" or Cool Tip or StarBurst or RITA or Acculis or Uniblate or Habib).tw,kw. | 171 |
| 50 | (Boston Scientific or Angiodynamics or Medtronic).tw,kw. | 1331 |
| 51 | or/49-50 [RF ABLATION DEVICES/SYSTEMS] | 1496 |
| 52 | exp Ablation Techniques/ | 5188 |
| 53 | ablat*.tw,kw. | 6715 |
| 54 | thermal heat*.tw,kw. | 32 |
| 55 | (thermal therap* or thermotherap* or thermo-therap*).tw,kw. | 404 |
| 56 | Electrocoagulation/ | 657 |
| 57 | coagulation*.tw,kw. | 6363 |
| 58 | (electrocoagulation* or electro-coagulation*).tw,kw. | 264 |
| 59 | (thermocoagulation* or thermo-coagulation*).tw,kw. | 124 |
| 60 | (electrocauter* or electro-cauter*).tw,kw. | 627 |
| 61 | or/52-60 [ABLATION/COAGULATION] | 16947 |
| 62 | 26 and 61 [MICROWAVES + ABLATION/COAGULATION] | 304 |
| 63 | 48 and 61 [RADIO WAVES + ABLATION/COAGULATION] | 2240 |
| 64 | 20 or 29 or 41 or 51 or 62 or 63 [MICROWAVE/RADIOFREQUENCY ABLATION] | 5323 |
| 65 | 6 and 64 [LUNG CANCER AND ABLATION] | 75 |
| 66 | exp Child/ not (exp Adult/ or Adolescent/) | 21936 |
| 67 | exp Infant/ not (exp Adult/ or Adolescent/) | 19554 |
| 68 | 65 not (66 or 67) [INFANT-, CHILD-ONLY REMOVED] | 75 |
| 69 | conference abstract.pt. | 43542 |
| 70 | 68 not 69 [CONFERENCE ABSTRACTS REMOVED] | 70 |
| 71 | limit 70 to yr="2005-current" | 65 |
| 72 | remove duplicates from 71 | 62 |

Table 4: Database(s): EBM Reviews - Cochrane Central Register of Controlled Trials December 2021, EBM Reviews - Cochrane Database of Systematic Reviews 2005 to January 12, 2022, Embase 1974 to 2022 January 14, Ovid MEDLINE(R) and Epub Ahead of Print, In-Process, In-Data-Review & Other Non-Indexed Citations and Daily 1946 to January 14, 2022 Search Strategy

Search Strategy:

| **#** | **Searches** | **Results** |
| --- | --- | --- |
| 1 | exp Lung Neoplasms/ or (((neoplas$ or cancer$ or tumour$ or tumor$ or carcinoma$ or malignan$ or metasta$ or oncolog$ or SCLC or NSCLC) adj3 (lung? or pulmonary or pleuropulmonary or bronch$)) or ((adenoma? or adenocarcinoma? or adeno-carcinoma? or blastoma? or carcinosarcoma? or carcino-sarcoma? or leukemia? or leukaemia? or lymphoma? or melanoma? or mesenchymoma? or mesothelioma? or sarcoma? or thymoma?) adj3 (lung$ or pulmonary or pleuropulmonary or bronch$)) or (Pancoast adj (tumor$ or tumour$ or syndrome$))).tw,kf. [LUNG CANCER] | 924748 |
| 2 | ((microwave$ adj3 (therap$ or treatment$)) or ((microwave or MW) adj2 (ablation$ or coagulation$)) or ((MCT or MCTs or MWA or PMCT or PMCTs or PMAT or PMATs or RALTCT or RALTCTs) adj5 (ablat$ or coagulat$ or postablat$ or post-ablat$ or thermocoagulat$ or thermo-coagulati$)) or ((microwave or MW) adj2 (ablat$ or coagulat$ or postablat$ or post-ablat$ or thermocoagulat$ or thermo-coagulati$)) or ((microwave or MW) adj2 (electrocoagulation$ or electro-coagulation$ or electrocauter$ or electro-cauter$ or thermal therap$ or thermotherap$ or thermo-therap$))).ti,ab,kw,kf. or ((Acculis$ or Amica$ or AMICA-GEM$ or Avecure$ or Certus$ or Emblation$ or Emprint$ or FORSEA$ or MicrothermX$ or MSYS245 or NEUWAVE$ or Solero$ or TATO or TATOPro$ or BSD-2000$ or BSD-500$ or "Brilliance CT" or "RITA 1500" or KY-2000) and (Evident$ adj4 Covidien)).tw,kf. [MICROWAVE ABLATION; MICROWAVE ABLATION DEVICES/SYSTEMS] | 12736 |
| 3 | Microwaves/ or (microwave$ or micro wave$ or ((EHF or "high frequency" or ultrahigh or ultra-high) adj2 (radiowave$ or radio wave$ or wave$))).tw,kf. or Radio waves/ or (((high frequency or HF) adj2 (current$ or radiowave$ or radio wave$ or wave$)) or Hertzian Wave$ or radiofrequency or Short Wave$).tw,kf. [MICROWAVES OR RADIO WAVES] | 212941 |
| 4 | exp Ablation Techniques/ or Electrocoagulation/ or (ablat$ or coagulat$ or postablat$ or post-ablat$ or thermocoagulat$ or thermo-coagulati$ or electrocoagulation$ or electro-coagulation$ or electrocauter$ or electro-cauter$ or thermal therap$ or thermotherap$ or thermo-therap$).tw,kf. [ABLATION/COAGULATION] | 712384 |
| 5 | 2 or (3 and 4) | 85803 |
| 6 | 1 and 5 | 3655 |
| 7 | randomized controlled trial/ or controlled clinical trial/ or (randomized or placebo or randomly or trial or groups).ab. or drug therapy.fs. [RANDOMIZED STUDIES – MEDLINE sensitive Filter – Cochrane Handbook, 2019] | 14294862 |
| 8 | Cohort studies/ or comparative study/ or follow-up studies/ or prospective studies/ or risk factors/ or cohort.mp. or compared.mp. or groups.mp. or multivariate.mp. [NON-RANDOMIZED STUDIES – MEDLINE Filter - sensitive, Furlan,2006] | 19531549 |
| 9 | Comparative studies/ or Follow-up studies/ or Time factors/ or (preoperat$ or pre operat$).mp. or (chang$ or evaluat$ or reviewed or prospective$ or retrospective$ or baseline or cohort or case series).tw. [OBSERVATIONAL STUDIES – MEDLINE Filter – max specificity, Fraser, 2006] | 24288285 |
| 10 | exp cohort studies/ or non-randomized controlled trials as topic/ or controlled before-after studies/ or interrupted time series analysis/ or historically controlled study/ or case-control studies/ or cross-sectional studies/ or comparative study/ or observational study/ or (((cohort or concurrent or non-concurrent or incidence or follow-up or followup or longitudinal or prospective or retrospective or nonrandom$ or non-random$ or quasi-random$ or quasi-experiment$ or non-RCT or nRCT or pretest or posttest or pre-test or post-test or "before after" or "CBA stud$" or "ITS stud$" or (historical$ adj2 control$) or case-control or case-comparison or case-compeer or case-referrent or case-base or cross-sectional or prevalence) adj3 (stud$ or design?)) or real-world or RWE or regist$ or (interrupted adj2 time adj2 series)).tw,kf. [ADDITIONAL TERMS TO SUPPLEMENT NRS FILTERS] | 10766707 |
| 11 | ("single arm" adj2 (stud$ or design?)).tw,kf. [SINGLE-ARM STUDIES – MEDLINE] | 12014 |
| 12 | (((Meta-Analysis as Topic/ or exp Review Literature as Topic/ or Meta-Analysis/ or (meta analy$ or metaanaly$ or (systematic adj (review$1 or overview$1))).tw. or (cochrane or embase or (psychlit or psyclit) or (psychinfo or psycinfo) or (cinahl or cinhal) or "science citation index" or bids or cancerlit or "reference list$" or bibliograph$ or "hand-search$" or "relevant journals" or "manual search$").ab. or (Review/ and (selection criteria or data extraction).ab.)) not Comment/) or Letter/ or Editorial/ or exp Animals/) not (exp Animals/ and Humans/) [Scottish Intercollegiate Guidelines Network (SIGN) SR and MA filter - specificity] | 17852965 |
| 13 | 7 or 8 or 9 or 10 or 11 or 12 | 44040577 |
| 14 | 6 and 13 | 3009 |
| 15 | 14 use ppez | 924 |
| 16 | exp Animals/ not Humans/ | 17078929 |
| 17 | 15 not 16 | 859 |
| 18 | (address or autobiography or bibliography or biography or comment or dictionary or directory or editorial or "expression of concern" or festschrift or historical article or interactive tutorial or lecture or legal case or legislation or news or newspaper article or patient education handout or personal narrative or portrait or video-audio media or webcast or (letter not (letter and randomized controlled trial))).pt. | 4593891 |
| 19 | 17 not 18 | 845 |
| 20 | limit 19 to dt="20210401-20221231" [Limit not valid in CCTR,CDSR,Embase; records were retained] | 51 |
| 21 | limit 20 to yr="2021 - Current" | 51 |
| 22 | lung tumor/ or exp lung cancer/ or lung adenoma/ or (((neoplas$ or cancer$ or tumour$ or tumor$ or carcinoma$ or malignan$ or metasta$ or oncolog$ or SCLC or NSCLC) adj3 (lung? or pulmonary or pleuropulmonary or bronch$)) or ((adenoma? or adenocarcinoma? or adeno-carcinoma? or blastoma? or carcinosarcoma? or carcino-sarcoma? or leukemia? or leukaemia? or lymphoma? or melanoma? or mesenchymoma? or mesothelioma? or sarcoma? or thymoma?) adj3 (lung$ or pulmonary or pleuropulmonary or bronch$)) or (Pancoast adj (tumor$ or tumour$ or syndrome$))).tw,kw. [LUNG CANCER] | 920909 |
| 23 | microwave ablation device/ or ((microwave$ adj3 (therap$ or treatment$)) or ((microwave or MW) adj2 (ablation$ or coagulation$)) or ((MCT or MCTs or MWA or PMCT or PMCTs or PMAT or PMATs or RALTCT or RALTCTs) adj5 (ablat$ or coagulat$ or postablat$ or post-ablat$ or thermocoagulat$ or thermo-coagulati$)) or ((microwave or MW) adj2 (ablat$ or coagulat$ or postablat$ or post-ablat$ or thermocoagulat$ or thermo-coagulati$)) or ((microwave or MW) adj2 (electrocoagulation$ or electro-coagulation$ or electrocauter$ or electro-cauter$ or thermal therap$ or thermotherap$ or thermo-therap$))).tw,kw. or ((Acculis$ or Amica$ or AMICA-GEM$ or Avecure$ or Certus$ or Emblation$ or Emprint$ or FORSEA$ or MicrothermX$ or MSYS245 or NEUWAVE$ or Solero$ or TATO or TATOPro$ or BSD-2000$ or BSD-500$ or "Brilliance CT" or "RITA 1500" or KY-2000) and (Evident$ adj4 Covidien)).tw,kw,dv. [MICROWAVE ABLATION; MICROWAVE ABLATION DEVICES/SYSTEMS] | 12487 |
| 24 | microwave thermotherapy/ or microwave radiation/ or (microwave$ or micro wave$ or ((EHF or "high frequency" or ultrahigh or ultra-high) adj2 (radiowave$ or radio wave$ or wave$))).tw,kw. or radiofrequency radiation/ or (((high frequency or HF) adj2 (current$ or radiowave$ or radio wave$ or wave$)) or Hertzian Wave$ or radiofrequency or Short Wave$).tw,kw. [MICROWAVES OR RADIO WAVES] | 211350 |
| 25 | ablation therapy/ or Electrocoagulation/ or radiofrequency ablation/ or (ablat$ or coagulat$ or postablat$ or post-ablat$ or thermocoagulat$ or thermo-coagulati$ or electrocoagulation$ or electro-coagulation$ or electrocauter$ or electro-cauter$ or thermal therap$ or thermotherap$ or thermo-therap$).tw,kw. [ABLATION/COAGULATION] | 632288 |
| 26 | 23 or (24 and 25) | 82591 |
| 27 | 22 and 26 | 3669 |
| 28 | Randomized controlled trial/ or Controlled clinical study/ or randomization/ or intermethod comparison/ or double blind procedure/ or human experiment/ or (compare or compared or comparison or trial).ti. or ((evaluated or evaluate or evaluating or assessed or assess) and (compare or compared or comparing or comparison)).ab. or (random$ or placebo or (open adj label) or ((double or single or doubly or singly) adj (blind or blinded or blindly)) or parallel group$1 or (crossover or cross over) or ((assign$ or match or matched or allocation) adj5 (alternate or group$1 or intervention$1 or patient$1 or subject$1 or participant$1)) or (assigned or allocated) or (controlled adj7 (study or design or trial)) or (volunteer or volunteers)).ti,ab. | 10642018 |
| 29 | (Cross-sectional study/ not (randomized controlled trial/ or controlled clinical study/ or controlled study/ or randomi?ed controlled.ti,ab. or control group$1.ti,ab.)) or ((((case adj control$) and random$) not randomi?ed controlled) or (nonrandom$ not random$) or "Random field$" or (random cluster adj3 sampl$)).ti,ab. or (Systematic review not (trial or study)).ti. or ((review.ab. and review.pt.) not trial.ti.) or ("we searched".ab. and (review.ti. or review.pt.)) or ("update review" or (databases adj4 searched)).ab. or ((rat or rats or mouse or mice or swine or porcine or murine or sheep or lambs or pigs or piglets or rabbit or rabbits or cat or cats or dog or dogs or cattle or bovine or monkey or monkeys or trout or marmoset$1).ti. and animal experiment/) or (Animal experiment/ not (human experiment/ or human/)) | 5429432 |
| 30 | 28 not 29 | 9724752 |
| 31 | Clinical article/ or controlled study/ or major clinical study/ or prospective study/ or cohort.mp. or compared.mp. or groups.mp. or multivariate.mp. [NON-RANDOMIZED STUDIES– Embase Filter - sensitive, Furlan,2006] | 23418303 |
| 32 | Controlled study/ or Treatment outcome/ or Major clinical study/ or Clinical trial/ or (chang$ or evaluat$ or reviewed or baseline or (compare$ or compara$)).tw. [Observational Studies – Embase Filter – max specificity, Fraser, 2006] | 31124653 |
| 33 | exp cohort analysis/ or exp case control study/ or controlled clinical trial/ or pretest posttest control group design/ or static group comparison/ or retrospective study/ or longitudinal study/ or intervention study/ or family study/ or case study/ or time series analysis/ or cross-sectional study/ or comparative study/ or observational study/ or quasi experimental study/ or (((cohort or concurrent or non-concurrent or incidence or follow-up or followup or longitudinal or prospective or retrospective or nonrandom$ or non-random$ or quasi-random$ or quasi-experiment$ or non-RCT or nRCT or pretest or posttest or pre-test or post-test or "before after" or "CBA stud$" or "ITS stud$" or (historical$ adj2 control$) or case-control or case-comparison or case-compeer or case-referrent or case-base or cross-sectional or prevalence) adj3 (stud$ or design?)) or real-world or RWE or regist$ or (interrupted adj2 time adj2 series)).tw,kw. [ADDITIONAL TERMS TO SUPPLEMENT NRS FILTERS] | 14055636 |
| 34 | ("single arm" adj2 (stud$ or design?)).tw,kw. [SINGLE-ARM STUDIES – MEDLINE] | 12003 |
| 35 | ((exp Meta Analysis/ or ((meta adj analy$) or metaanalys$ or (systematic adj (review$1 or overview$1))).tw. or (cancerlit or cochrane or embase or (psychlit or psyclit) or (psychinfo or psycinfo) or (cinahl or cinhal) or science citation index or bids).ab. or (reference lists or bibliograph$ or hand-search$ or manual search$ or relevant journals).ab. or ((data extraction or selection criteria).ab. and review.pt.)) not letter.pt.) or editorial.pt. or ((exp animal experimentation/ or exp animal model/ or exp animal experiment/ or nonhuman/ or exp vertebrate/) not (exp human/ or exp human experimentation/ or exp human experiment/)) [Scottish Intercollegiate Guidelines Network (SIGN) SR and MA filter - specificity] | 12919371 |
| 36 | 30 or 31 or 32 or 33 or 34 or 35 | 45057873 |
| 37 | 27 and 36 | 2968 |
| 38 | 37 use oemezd | 1784 |
| 39 | exp animal experimentation/ or exp animal model/ or exp animal experiment/ or nonhuman/ or exp vertebrate/ | 54480887 |
| 40 | exp human/ or exp human experimentation/ or exp human experiment/ | 43842478 |
| 41 | 38 not (39 not 40) | 1698 |
| 42 | (editorial or letter or note or short survey or tombstone).pt. | 4929975 |
| 43 | 41 not 42 | 1656 |
| 44 | limit 43 to dd="20210401-20221231" [Limit not valid in CCTR,CDSR,Ovid MEDLINE(R),Ovid MEDLINE(R) Daily Update,Ovid MEDLINE(R) PubMed not MEDLINE,Ovid MEDLINE(R) In-Process,Ovid MEDLINE(R) Publisher; records were retained] | 42 |
| 45 | limit 44 to yr="2021 - Current" | 37 |
| 46 | exp Lung Neoplasms/ or (((neoplas$ or cancer$ or tumour$ or tumor$ or carcinoma$ or malignan$ or metasta$ or oncolog$ or SCLC or NSCLC) adj3 (lung? or pulmonary or pleuropulmonary or bronch$)) or ((adenoma? or adenocarcinoma? or adeno-carcinoma? or blastoma? or carcinosarcoma? or carcino-sarcoma? or leukemia? or leukaemia? or lymphoma? or melanoma? or mesenchymoma? or mesothelioma? or sarcoma? or thymoma?) adj3 (lung$ or pulmonary or pleuropulmonary or bronch$)) or (Pancoast adj (tumor$ or tumour$ or syndrome$))).tw,kw. [LUNG CANCER] | 924106 |
| 47 | ((microwave$ adj3 (therap$ or treatment$)) or ((microwave or MW) adj2 (ablation$ or coagulation$)) or ((MCT or MCTs or MWA or PMCT or PMCTs or PMAT or PMATs or RALTCT or RALTCTs) adj5 (ablat$ or coagulat$ or postablat$ or post-ablat$ or thermocoagulat$ or thermo-coagulati$)) or ((microwave or MW) adj2 (ablat$ or coagulat$ or postablat$ or post-ablat$ or thermocoagulat$ or thermo-coagulati$)) or ((microwave or MW) adj2 (electrocoagulation$ or electro-coagulation$ or electrocauter$ or electro-cauter$ or thermal therap$ or thermotherap$ or thermo-therap$)) or ((Acculis$ or Amica$ or AMICA-GEM$ or Avecure$ or Certus$ or Emblation$ or Emprint$ or FORSEA$ or MicrothermX$ or MSYS245 or NEUWAVE$ or Solero$ or TATO or TATOPro$ or BSD-2000$ or BSD-500$ or "Brilliance CT" or "RITA 1500" or KY-2000) and (Evident$ adj4 Covidien))).tw,kw. [MICROWAVE ABLATION; MICROWAVE ABLATION DEVICES/SYSTEMS] | 12457 |
| 48 | Microwaves/ or (microwave$ or micro wave$ or ((EHF or "high frequency" or ultrahigh or ultra-high) adj2 (radiowave$ or radio wave$ or wave$))).tw,kw. or Radio waves/ or (((high frequency or HF) adj2 (current$ or radiowave$ or radio wave$ or wave$)) or Hertzian Wave$ or radiofrequency or Short Wave$).tw,kw. [MICROWAVES OR RADIO WAVES] | 210615 |
| 49 | exp Ablation Techniques/ or Electrocoagulation/ or (ablat$ or coagulat$ or postablat$ or post-ablat$ or thermocoagulat$ or thermo-coagulati$ or electrocoagulation$ or electro-coagulation$ or electrocauter$ or electro-cauter$ or thermal therap$ or thermotherap$ or thermo-therap$).tw,kw. [ABLATION/COAGULATION] | 702087 |
| 50 | 47 or (48 and 49) | 83598 |
| 51 | 46 and 50 | 3609 |
| 52 | 51 use cctr | 103 |
| 53 | 52 and ((2021* not (202101* or 202102* or 202103*)) or 2022*).up. | 26 |
| 54 | limit 53 to yr="2021 - Current" | 13 |
| 55 | (((neoplas$ or cancer$ or tumour$ or tumor$ or carcinoma$ or malignan$ or metasta$ or oncolog$ or SCLC or NSCLC) adj3 (lung? or pulmonary or pleuropulmonary or bronch$)) or ((adenoma? or adenocarcinoma? or adeno-carcinoma? or blastoma? or carcinosarcoma? or carcino-sarcoma? or leukemia? or leukaemia? or lymphoma? or melanoma? or mesenchymoma? or mesothelioma? or sarcoma? or thymoma?) adj3 (lung$ or pulmonary or pleuropulmonary or bronch$)) or (Pancoast adj (tumor$ or tumour$ or syndrome$))).tw,kw. [LUNG CANCER] | 741789 |
| 56 | ((microwave$ adj3 (therap$ or treatment$)) or ((microwave or MW) adj2 (ablation$ or coagulation$)) or ((MCT or MCTs or MWA or PMCT or PMCTs or PMAT or PMATs or RALTCT or RALTCTs) adj5 (ablat$ or coagulat$ or postablat$ or post-ablat$ or thermocoagulat$ or thermo-coagulati$)) or ((microwave or MW) adj2 (ablat$ or coagulat$ or postablat$ or post-ablat$ or thermocoagulat$ or thermo-coagulati$)) or ((microwave or MW) adj2 (electrocoagulation$ or electro-coagulation$ or electrocauter$ or electro-cauter$ or thermal therap$ or thermotherap$ or thermo-therap$)) or ((Acculis$ or Amica$ or AMICA-GEM$ or Avecure$ or Certus$ or Emblation$ or Emprint$ or FORSEA$ or MicrothermX$ or MSYS245 or NEUWAVE$ or Solero$ or TATO or TATOPro$ or BSD-2000$ or BSD-500$ or "Brilliance CT" or "RITA 1500" or KY-2000) and (Evident$ adj4 Covidien))).tw,kw. [MICROWAVE ABLATION; MICROWAVE ABLATION DEVICES/SYSTEMS] | 12457 |
| 57 | (microwave$ or micro wave$ or ((EHF or "high frequency" or ultrahigh or ultra-high) adj2 (radiowave$ or radio wave$ or wave$)) or (((high frequency or HF) adj2 (current$ or radiowave$ or radio wave$ or wave$)) or Hertzian Wave$ or radiofrequency or Short Wave$)).tw,kw. [MICROWAVES OR RADIO WAVES] | 197904 |
| 58 | (ablat$ or coagulat$ or postablat$ or post-ablat$ or thermocoagulat$ or thermo-coagulati$ or electrocoagulation$ or electro-coagulation$ or electrocauter$ or electro-cauter$ or thermal therap$ or thermotherap$ or thermo-therap$).tw,kw. [ABLATION/COAGULATION] | 610781 |
| 59 | 56 or (57 and 58) | 80158 |
| 60 | 55 and 59 | 3218 |
| 61 | 60 use coch | 9 |
| 62 | 61 and ((2021* not (202101* or 202102* or 202103*)) or 2022*).up. | 3 |
| 63 | limit 62 to yr="2021 - Current" | 0 |
| 64 | 21 or 45 or 54 or 63 | 101 |
| **65** | **remove duplicates from 64** | **86** |

# Detailed PICOS and Additional Data Extraction Information

Table 5: PICOS criteria used to screen pulmonary metastases records

| **Population** | - Adults (≥18 years) with pulmonary metastases - Included patient threshold of ≥40 patients - Include patients with additional active sites of disease outside the lung if those sites were treated with MWA, RFA, or SBRT. |
| --- | --- |
| **Intervention** | - MWA or RFA or SBRT - Exclude studies that used the intervention as salvage therapy - Include combination treatments (e.g. MWA + chemo) - Studies on patients receiving first-line treatments or treatments for recurrence were included. - Include only specific SBRT modalities**:** i.e., X-ray photon only - Include MWA and RFA studies that use the percutaneous approach only |
| **Outcomes** | - 1, 2, and 3-year OS - 1, 2, and 3-year LTP - For patients with additional active sites of disease outside the lung, extract lung specific LTP only. |
| **Study Design** | RCTs, Comparative Observational, Single-arm studies, and Single-arms from comparative studies |
| **Language** | English only |
| **Date Restrictions** | Jan 1, 2005 to January 16, 2022 |

Abbreviations: LTP=local tumor progression; MWA=microwave ablation; OS=overall survival; PICOS=Population, Intervention, Comparison, Outcomes and Study Design; RCT=randomized controlled trial; RFA=radiofrequency ablation; SBRT=stereotactic body radiation therapy.

**Data Extraction**

Multiple study arms were extracted from a study if they reported different treatments of interest (e.g., comparative study of MWA vs. RFA) and pulmonary metastases-only data was extracted from studies that included both NSCLC and patients with pulmonary metastases.

# Quality Assessment of Studies (MINORS)

Table 6: Study evaluation heuristics for questions one through eight of the MINORS tool

| **Criteria** | **Guidelines from Original Article** | **Scored 0/2** | **Scored 1/2** | **Scored 2/2** |
| --- | --- | --- | --- | --- |
| **Criterion 1: A clearly stated aim** | “The question addressed should be precise and relevant in the light of available literature” | Aim/objective of study is missing | Aim/objective of study is unclear/undefined | Aim/objective of study is clearly defined (aim does not need to be relevant to our study) |
| **Criterion 2: Inclusion of consecutive patients** | “All patients potentially fit for inclusion (satisfying the criteria for inclusion) have been included in the study during the study period (no exclusion or details about the reasons for exclusion)” | Inclusion criteria is missing AND patients are not consecutive | Inclusion criteria is unclear/undefined OR patients are not explicitly mentioned as consecutive/it is unclear | Inclusion criteria for patients is clearly defined (exclusion criteria not necessary) AND patients are consecutive |
| **Criterion 3: Prospective collection of data** | “Data were collected according to a protocol established before the beginning of the study” | Study is unclear regarding design or truly retrospective with no follow-up (case control, etc.) | Study design is retrospective cohort study | Study design is prospective, with protocol established before beginning of study |
| **Criterion 4: Endpoints appropriate to the aim of the study** | “Unambiguous explanation of the criteria used to evaluate the main outcome, which should be in accordance with the question addressed by the study. Also, the endpoints should be assessed on an intention-to-treat basis.” | Endpoint definition for LTP and DFS missing or unclear | Endpoint definition for only one of LTP or DFS is included and clear | Endpoint definitions for LTP and DFS are included and clear. DFS includes death as an event, LTP definition must include method used to assess LTP (CT scan, PET scan, etc.) |
| **Criterion 5: Unbiased assessment of the study endpoint** | “Blind evaluation of objective endpoints and double-blind evaluation of subjective  endpoints. Otherwise, the reasons for not blinding should be stated.” | Outcomes of focus are subjective OR objective and not quantifiable, and there is no blinding (subjective outcomes include PROs and QoL assessments, etc.) | Unclear risk of bias -- no blinding but objective outcome is quantifiable (e.g., LTP is defined as progression 1 cm outside of the tumour). | Study uses an a-priori protocol or criteria, an external tumor board or group of reviewers, or RECIST criteria to determine progression or outcomes, or study applies blinding methods (e.g., external assessor) |
| **Criterion 6: Follow-up period appropriate to the aim of the study*** | “The follow-up should be sufficiently long to allow the assessment of the main endpoint and possible adverse events.” | Planned or median follow-up period is less than 2 years | Planned or median follow-up period is 2-3 years | Planned or median follow-up period is at least 3 years |
| **Criterion 7: Loss to follow up less than 5%*** | “All patients should be included in the follow up. Otherwise, the proportion lost to follow up should not exceed the proportion experiencing the major endpoint.” | Loss to follow up is not reported (does not include patients who died) | Loss to follow-up is unclear or >5% | Loss to follow up is reported and <5% |
| **Criterion 8: Prospective calculation of the study size** | “Information of the size of detectable difference of interest with a calculation of  95% confidence interval, according to the expected incidence of the outcome event, and information about the level for  statistical significance and estimates of power when comparing the outcomes.” | Prospective calculation of study size is not reported | Prospective calculation of study size is unclear/undefined | Prospective calculation of study size is reported (most studies not expected to achieve 2/2 on this criteria) |

Abbreviations: CT=computed tomography; DFS=disease-free survival; LTP=local tumor progression; PET=positron emission tomography; PROs=patient-reported outcomes; QoL=quality of life; RECIST=[Response Evaluation Criteria in Solid Tumors](https://ctep.cancer.gov/protocoldevelopment/docs/quickrcst.doc#:~:text=Each%20patient%20will%20be%20assigned,not%20assessable%2C%20insufficient%20data).).

# Proportion of Primary Tumor Locations

The following table describes the proportion of patients with various primary tumor locations across studies included in the analyses. The primary tumor locations were well reported among the study arms as 77% and 82% of IGTA and SBRT studies, respectively, reported the types of primary tumors included.

Table 7: The proportion of patients with various primary tumor locations

| Primary tumor location | Treatment Arm^a^ | |
| --- | --- | --- |
|  | IGTA (n = 27/35) | SBRT (n = 47/57) |
| Colorectal carcinoma | 63.92% | 43.19% |
| Renal cell carcinoma | 6.95% | 4.00% |
| Sarcoma cancer | 6.48% | 4.09% |
| Pancreas/Kidney cancer | 0.65% | 0.08% |
| Breast cancer | 3.01% | 3.82% |
| Gynecologic cancers^b^ | 0.60% | 1.10% |
| Thyroid cancer | 1.39% | 0.06% |
| Head/neck cancer | 0.14% | 2.94% |
| Lung cancer | 1.62% | 19.81% |
| Bronchial cancer | 1.16% | 0.00% |
| Prostate cancer | 1.02% | 0.04% |
| Melanoma | 0.42% | 2.17% |
| Esophageal cancer | 0.88% | 1.00% |
| Gastric cancer | 1.07% | 1.83% |
| Liver | 2.73% | 1.09% |
| Unknown cancer | 0.09% | 0.0% |
| Other^c^ | 7.83% | 14.75% |
| Missing | 0.05% | 0.04% |

Footnotes: a The percentages are calculated based on the studies that reported primary tumor locations (n = 27 for IGTA and n = 47 for SBRT). The total number of patients with each primary tumor location was divided by the total number of patients among studies that reported the primary tumor location. b Includes cervical cancer, ovarian cancer, vaginal cancer, and vulvar cancer. c Other includes cancer types designated as “other” within the publication or includes a cancer type not already captured in the table (eg, ureter cancer or bladder cancer).

Abbreviations: IGTA = image-guided tumor ablation; SBRT=stereotactic body radiation therapy.

# Patients with Pulmonary Metastases Meta-Regressions

## Patients with Pulmonary Metastases Tables for Univariable Analyses for LTP and OS

Table 8: Patients with Pulmonary Metastases LTP Univariable Analyses for Treatment Covariate

|  | covariate | Estimated OR | | 95% CI OR | | P-value | |
| --- | --- | --- | --- | --- | --- | --- | --- |
| **Comparison to IGTA** | | | | | | | |
| 1-year LTP | **Treatment: SBRT** | | **0.629** | | **(0.424, 0.933)** | | **0.021** |
| 2-year LTP | Treatment: SBRT | | 1.200 | | (0.783, 1.84) | | 0.403 |
| 3-year LTP | **Treatment: SBRT** | | **1.693** | | **(1.050, 2.729)** | | **0.031** |

Abbreviations: CI=confidence interval; IGTA=image-guided thermal ablation; LTP=local tumor progression; OR=odds ratio; SBRT=stereotactic body radiation therapy.

Table 9: Patients with Pulmonary Metastases OS Univariable Analyses for Treatment Covariate

|  | covariate | Estimated OR | | 95% CI OR | | P-value | |
| --- | --- | --- | --- | --- | --- | --- | --- |
| **Comparison to IGTA** | | | | | | | |
| 1-year OS | Treatment: SBRT | | 0.787 | | (0.546, 1.134) | | 0.198 |
| 2-year OS | Treatment: SBRT | | 0.862 | | (0.607, 1.222) | | 0.404 |
| 3-year OS | Treatment: SBRT | | 0.984 | | (0.71, 1.362) | | 0.920 |

Abbreviations: CI=confidence interval; IGTA=image-guided thermal ablation; OR=odds ratio; OS=overall survival; SBRT=stereotactic body radiation therapy.

# Heat-Maps for Study-Level Covariates That Are Associated with LTP and OS

Figure 1: Study-level covariates associated with LTP or OS in multivariable meta regressions


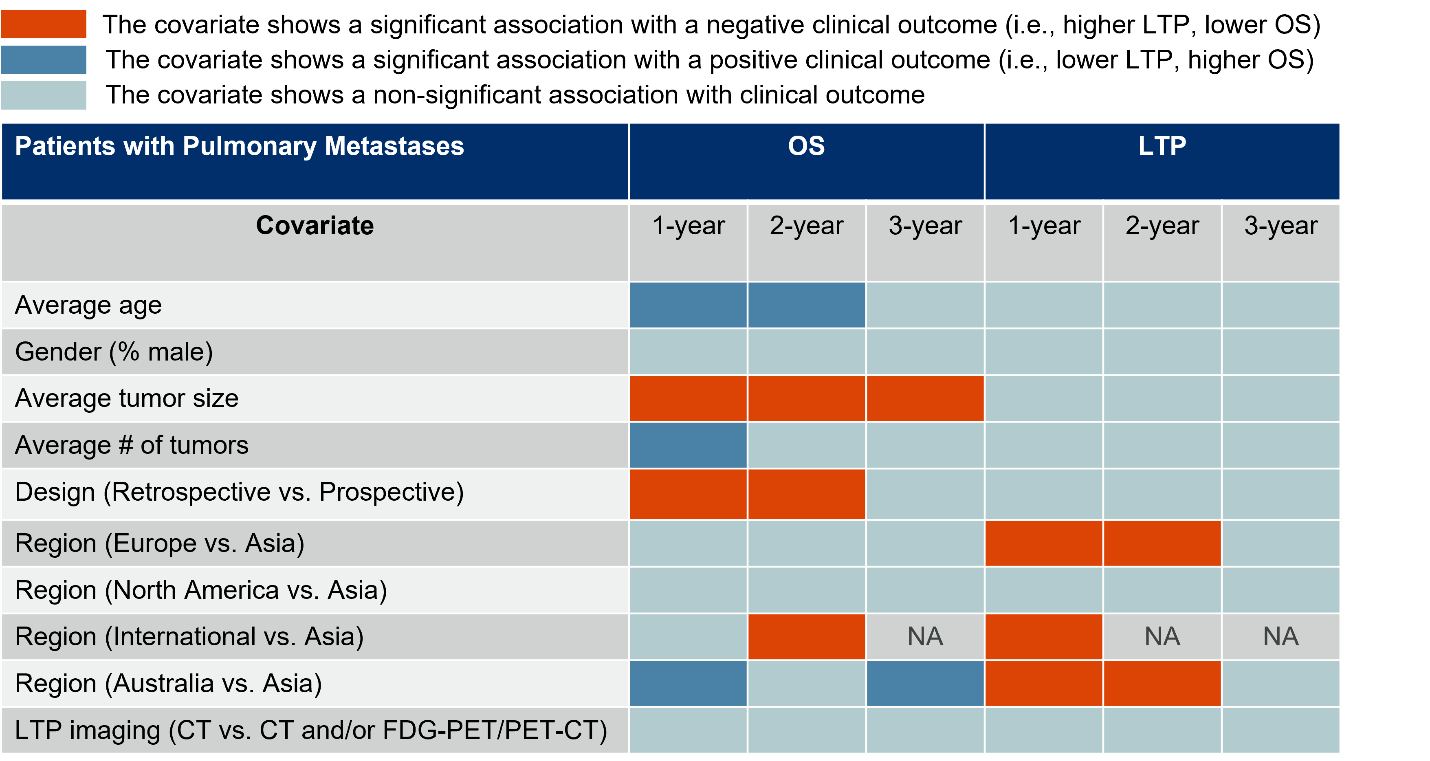


Abbreviations: CT=computed tomography; FDG=fluorodeoxyglucose; LTP=local tumor progression; NA=not applicable; OS=overall survival; PET=positron emission tomography; SBRT=stereotactic body radiation therapy.

#

# MINORS Assessment of Studies

Table 10: MINORS Assessment Scores per Criterion for Each Included Study

| **Author, year** | **Treatment** | **Criterion 1** | **Criterion 2** | **Criterion 3** | **Criterion 4** | **Criterion 5** | **Criterion 6** | **Criterion 7** | **Criterion 8** | **Total** |
| --- | --- | --- | --- | --- | --- | --- | --- | --- | --- | --- |
| Agolli, 2017 | SABR | 2 | 1 | 1 | 1 | 2 | 2 | 0 | 0 | 9 |
| Ahmed, 2018 | SBRT | 2 | 2 | 1 | 2 | 2 | 0 | 0 | 0 | 9 |
| Aitken, 2015 | SBRT | 2 | 1 | 1 | 2 | 0 | 0 | 0 | 0 | 6 |
| Akhan, 2016 | RFA | 2 | 1 | 1 | 2 | 0 | 1 | 0 | 0 | 7 |
| Ambrogi, 2006 | RFA | 2 | 1 | 2 | 1 | 2 | 1 | 0 | 0 | 9 |
| Aoki, 2016 | SBRT | 2 | 1 | 1 | 2 | 0 | 1 | 0 | 0 | 7 |
| Bae, 2012 | SBRT | 2 | 1 | 1 | 0 | 0 | 1 | 0 | 0 | 5 |
| Baumann, 2018 | SBRT | 2 | 1 | 1 | 2 | 0 | 0 | 0 | 0 | 6 |
| Benson, 2021 | SBRT | 2 | 1 | 1 | 2 | 0 | 1 | 1 | 0 | 8 |
| Berkovic, 2020 | SBRT | 2 | 2 | 1 | 2 | 2 | 0 | 0 | 0 | 9 |
| Binkley, 2015 | SBRT | 2 | 1 | 1 | 2 | 0 | 0 | 0 | 0 | 6 |
| Bonichon, 2013 | RFA | 2 | 1 | 2 | 2 | 0 | 1 | 1 | 0 | 9 |
| Chen, 2017 | RFA | 2 | 1 | 1 | 0 | 2 | 1 | 0 | 0 | 7 |
| Chua, 2010 | RFA | 2 | 1 | 1 | 2 | 2 | 0 | 0 | 0 | 8 |
| Comito, 2014 | SABR | 2 | 2 | 2 | 1 | 2 | 1 | 0 | 0 | 10 |
| De Baere, 2015 | RFA | 2 | 2 | 1 | 1 | 0 | 1 | 0 | 0 | 7 |
| Eriguchi, 2021 | SBRT | 2 | 1 | 1 | 2 | 0 | 1 | 0 | 0 | 7 |
| Fanucchi, 2016 | RFA | 2 | 1 | 2 | 1 | 2 | 1 | 2 | 0 | 11 |
| Ferguson, 2015 | RFA | 2 | 1 | 1 | 1 | 0 | 1 | 0 | 0 | 6 |
| Filippi, 2014 | SABR | 2 | 2 | 1 | 2 | 0 | 1 | 0 | 0 | 8 |
| Filippi, 2015 | SABR | 2 | 2 | 1 | 2 | 0 | 0 | 0 | 0 | 7 |
| Franceschini, 2017 | SBRT | 2 | 1 | 1 | 0 | 2 | 1 | 0 | 0 | 7 |
| Garcia-Cabezas, 2015 | SBRT | 2 | 2 | 1 | 1 | 2 | 0 | 0 | 0 | 8 |
| Geary, 2021 | SABR | 2 | 2 | 1 | 2 | 0 | 0 | 0 | 0 | 7 |
| Gonnet, 2019 | RFA | 2 | 2 | 1 | 2 | 1 | 2 | 0 | 0 | 10 |
| Guckenberger, 2009 | SBRT | 2 | 1 | 1 | 2 | 0 | 0 | 0 | 0 | 6 |
| Hasegawa, 2020 | RFA | 2 | 1 | 2 | 2 | 1 | 2 | 0 | 0 | 10 |
| Helou, 2017 | SABR | 2 | 2 | 1 | 2 | 1 | 0 | 0 | 0 | 8 |
| Hiyoshi, 2019 | RFA | 2 | 1 | 1 | 1 | 0 | 1 | 0 | 0 | 6 |
| Hof, 2007 | SBRT | 1 | 2 | 2 | 1 | 1 | 0 | 0 | 0 | 1 |
| Huang, 2011 | RFA | 2 | 2 | 1 | 1 | 2 | 1 | 0 | 2 | 11 |
| Inoue, 2013 | SBRT | 2 | 1 | 1 | 0 | 0 | 2 | 0 | 0 | 6 |
| Jang, 2017 | SBRT | 2 | 1 | 1 | 2 | 0 | 0 | 0 | 0 | 6 |
| Janssen, 2016 | SBRT | 2 | 1 | 1 | 0 | 0 | 2 | 0 | 0 | 6 |
| Jingu, 2017 | SBRT | 2 | 1 | 1 | 0 | 0 | 1 | 0 | 0 | 5 |
| Jung, 2015 | SABR | 2 | 1 | 1 | 1 | 2 | 2 | 0 | 0 | 9 |
| Kessel, 2020 | SBRT | 2 | 2 | 1 | 1 | 0 | 0 | 1 | 0 | 7 |
| Kinj, 2017 | SBRT | 2 | 1 | 1 | 2 | 2 | 1 | 0 | 0 | 9 |
| Kodama, 2015 | RFA | 2 | 1 | 2 | 2 | 0 | 0 | 0 | 0 | 7 |
| Korzets Cedar, 2018 | SBRT | 2 | 2 | 1 | 2 | 0 | 0 | 0 | 0 | 7 |
| Kurilova, 2018 | MWA | 2 | 1 | 1 | 0 | 0 | 1 | 1 | 0 | 6 |
| Lancia, 2019 | SBRT | 2 | 1 | 1 | 1 | 2 | 1 | 0 | 0 | 8 |
| Lencioni, 2008 | RFA | 2 | 1 | 2 | 1 | 0 | 0 | 1 | 2 | 9 |
| Lindsay, 2018 | SBRT | 2 | 1 | 1 | 0 | 0 | 0 | 0 | 0 | 4 |
| Lodeweges, 2017 | SABR | 2 | 2 | 1 | 0 | 0 | 2 | 0 | 0 | 7 |
| Loi, 2020 | SBRT | 2 | 2 | 1 | 2 | 2 | 0 | 0 | 0 | 9 |
| Matsui, 2015 | RFA | 2 | 1 | 1 | 0 | 0 | 2 | 0 | 0 | 6 |
| Mazzola, 2018 | SBRT | 2 | 1 | 1 | 1 | 2 | 0 | 0 | 0 | 7 |
| Mazzola, 2019 | SBRT | 2 | 1 | 2 | 0 | 2 | 0 | 0 | 0 | 7 |
| Najafi, 2020 | RFA | 2 | 2 | 1 | 0 | 2 | 2 | 0 | 0 | 9 |
| Navarria, 2014 | SBRT | 2 | 2 | 2 | 2 | 1 | 0 | 0 | 0 | 9 |
| Nicosia, 2021 | SABR | 2 | 1 | 1 | 2 | 0 | 1 | 1 | 0 | 8 |
| Nour-Eldin, 2017 | MWA and RFA | 2 | 2 | 1 | 0 | 2 | 2 | 0 | 0 | 9 |
| Nuyttens, 2012 | SBRT | 2 | 1 | 2 | 2 | 1 | 0 | 0 | 0 | 8 |
| Oh, 2012 | SBRT | 2 | 1 | 1 | 1 | 0 | 0 | 0 | 0 | 5 |
| Okunieff, 2006 | SBRT | 2 | 1 | 2 | 2 | 1 | 0 | 2 | 0 | 10 |
| Omae, 2016 | RFA | 2 | 1 | 1 | 2 | 0 | 2 | 0 | 0 | 8 |
| Osti, 2013 | SBRT | 2 | 1 | 2 | 2 | 0 | 0 | 0 | 0 | 7 |
| Osti, 2018 | SBRT | 2 | 1 | 1 | 2 | 1 | 2 | 0 | 0 | 9 |
| Parker, 2019 | SBRT | 2 | 2 | 1 | 1 | 2 | 0 | 0 | 0 | 8 |
| Pennathur, 2009 | RFA | 2 | 2 | 1 | 2 | 2 | 0 | 0 | 0 | 9 |
| Picchi, 2020 | RFA | 2 | 2 | 1 | 1 | 1 | 1 | 0 | 0 | 8 |
| Ricardi, 2012 | SBRT | 2 | 1 | 1 | 2 | 0 | 0 | 0 | 0 | 6 |
| Ricco, 2017 | SBRT | 2 | 1 | 1 | 2 | 2 | 0 | 0 | 0 | 8 |
| Rieber, 2016 | SBRT | 2 | 1 | 1 | 1 | 0 | 0 | 0 | 0 | 5 |
| Sato, 2016 | RFA | 2 | 1 | 1 | 2 | 0 | 0 | 0 | 0 | 6 |
| Scorsetti, 2015 | SBRT | 2 | 1 | 1 | 2 | 2 | 0 | 0 | 0 | 8 |
| Sharma, 2018 | SBRT | 2 | 1 | 1 | 2 | 2 | 0 | 0 | 0 | 8 |
| Sharma, 2019 | SBRT | 2 | 1 | 1 | 1 | 0 | 1 | 0 | 0 | 6 |
| Simon, 2007 | RFA | 2 | 2 | 1 | 2 | 0 | 0 | 0 | 0 | 7 |
| Siva, 2015 | SBRT | 2 | 2 | 1 | 2 | 2 | 1 | 0 | 0 | 10 |
| Siva, 2021 | SABR | 2 | 1 | 2 | 0 | 0 | 2 | 2 | 0 | 9 |
| Takahaski, 2012 | SBRT | 2 | 1 | 1 | 2 | 1 | 0 | 0 | 0 | 7 |
| Takeda, 2011 | SBRT | 2 | 1 | 1 | 0 | 0 | 0 | 0 | 0 | 4 |
| Tekatli, 2017 | SBRT | 2 | 2 | 1 | 0 | 0 | 1 | 0 | 0 | 6 |
| Vogl, 2018 | MWA | 2 | 1 | 1 | 0 | 2 | 0 | 0 | 0 | 6 |
| Vogl, 2016 | MWA and RFA | 2 | 1 | 1 | 0 | 0 | 1 | 1 | 0 | 6 |
| Von Meyenfeldt, 2011 | RFA | 2 | 2 | 1 | 1 | 0 | 0 | 0 | 0 | 6 |
| Wang, 2015 | RFA | 2 | 1 | 2 | 1 | 2 | 2 | 1 | 0 | 11 |
| Wang, 2015 | SBRT | 2 | 1 | 1 | 1 | 2 | 0 | 0 | 0 | 7 |
| Yamakado, 2007 | RFA | 2 | 1 | 1 | 2 | 0 | 0 | 0 | 0 | 6 |
| Yamakado, 2009 | RFA | 2 | 2 | 1 | 2 | 0 | 1 | 2 | 0 | 10 |
| Yamamoto, 2014 | SBRT | 2 | 1 | 1 | 2 | 1 | 1 | 0 | 0 | 8 |
| Yamashita, 2016 | SBRT | 2 | 1 | 1 | 0 | 0 | 0 | 0 | 0 | 4 |
| Yan, 2007 | RFA | 2 | 1 | 2 | 1 | 2 | 1 | 0 | 0 | 9 |
| Zhang, 2011 | SBRT | 2 | 1 | 1 | 1 | 2 | 1 | 2 | 0 | 10 |
| Zheng, 2016 | MWA | 2 | 2 | 1 | 1 | 2 | 1 | 2 | 0 | 11 |
| Zhong, 2020 | RFA | 2 | 2 | 1 | 2 | 0 | 2 | 0 | 0 | 9 |

Criterion 1: a clearly stated claim. Criterion 2: inclusion of consecutive patients. Criterion 3: prospective collection of data. Criterion 4: endpoints appropriate to the aim of the study. Criterion 5: unbiased assessment of the study endpoint. Criterion 6: follow-up period appropriate to the aim of the study. Criterion 7: Loss to follow up less than 5%. Criterion 8: prospective calculation of the study size.

Abbreviations: MWA = microwave ablation; RFA = radiofrequency ablation; SABR = stereotactic ablative radiotherapy; SBRT = stereotactic body radiation therapy.

# Publication Bias

Table 11: Patients with Pulmonary Metastases LFK Index and Egger’s Test for Publication Bias

| **Outcome** | **Timepoint** | **Treatment** | **P-value (Egger’s Test)** | **LFK index** |
| --- | --- | --- | --- | --- |
| LTP | 1-year | IGTA | 0.5698 | 0.84 |
|  |  | SBRT | 0.0007 | -2.71 |
|  | 2-year | IGTA | 0.6179 | -1.25 |
|  |  | SBRT | 0.0008 | -2.63 |
|  | 3-year | IGTA | 0.1714 | 1.95 |
|  |  | SBRT | 0.0080 | -2.20 |
| OS | 1-year | IGTA | 0.0895 | 1.58 |
|  |  | SBRT | <.0001 | 5.22 |
|  | 2-year | IGTA | 0.7872 | 0.12 |
|  |  | SBRT | <.0001 | 2.82 |
|  | 3-year | IGTA | 0.0563 | -2.22 |
|  |  | SBRT | 0.0006 | 1.73 |

Abbreviations: IGTA=image-guided thermal ablation; LFK=Luis Furuya-Kanamori; LTP=local tumor progression; OS=overall survival; SBRT=stereotactic body radiation therapy.

Figure 2: A) 1-year, B) 2-year, C) 3-year LTP Funnel Plots for Publication Bias


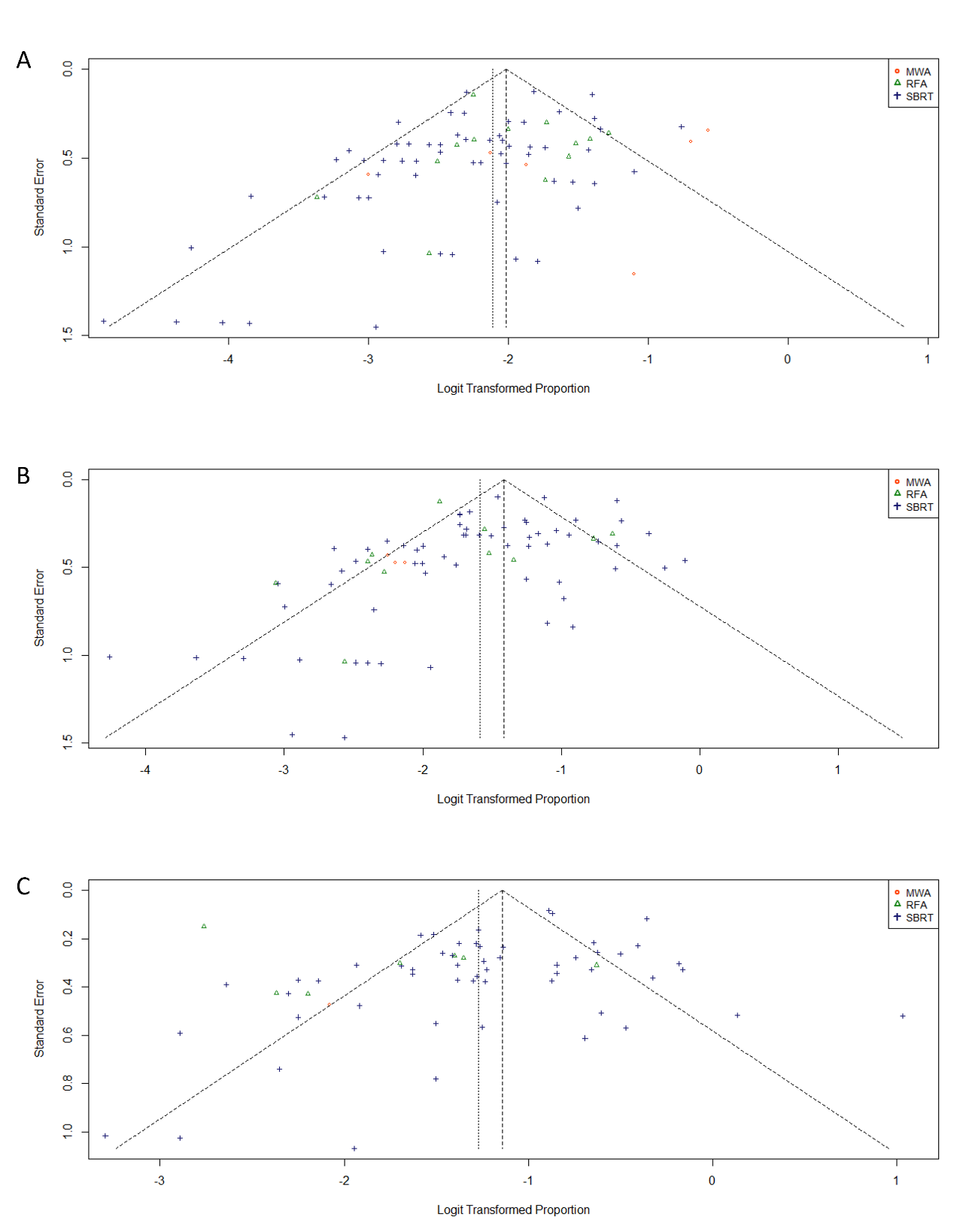


Abbreviations: LTP=local tumor progression; MWA=microwave ablation; RFA=radiofrequency ablation; SBRT=stereotactic body radiation therapy.

Figure 3: A) 1-year, B) 2-year, C) 3-year OS Funnel Plots for Publication Bias


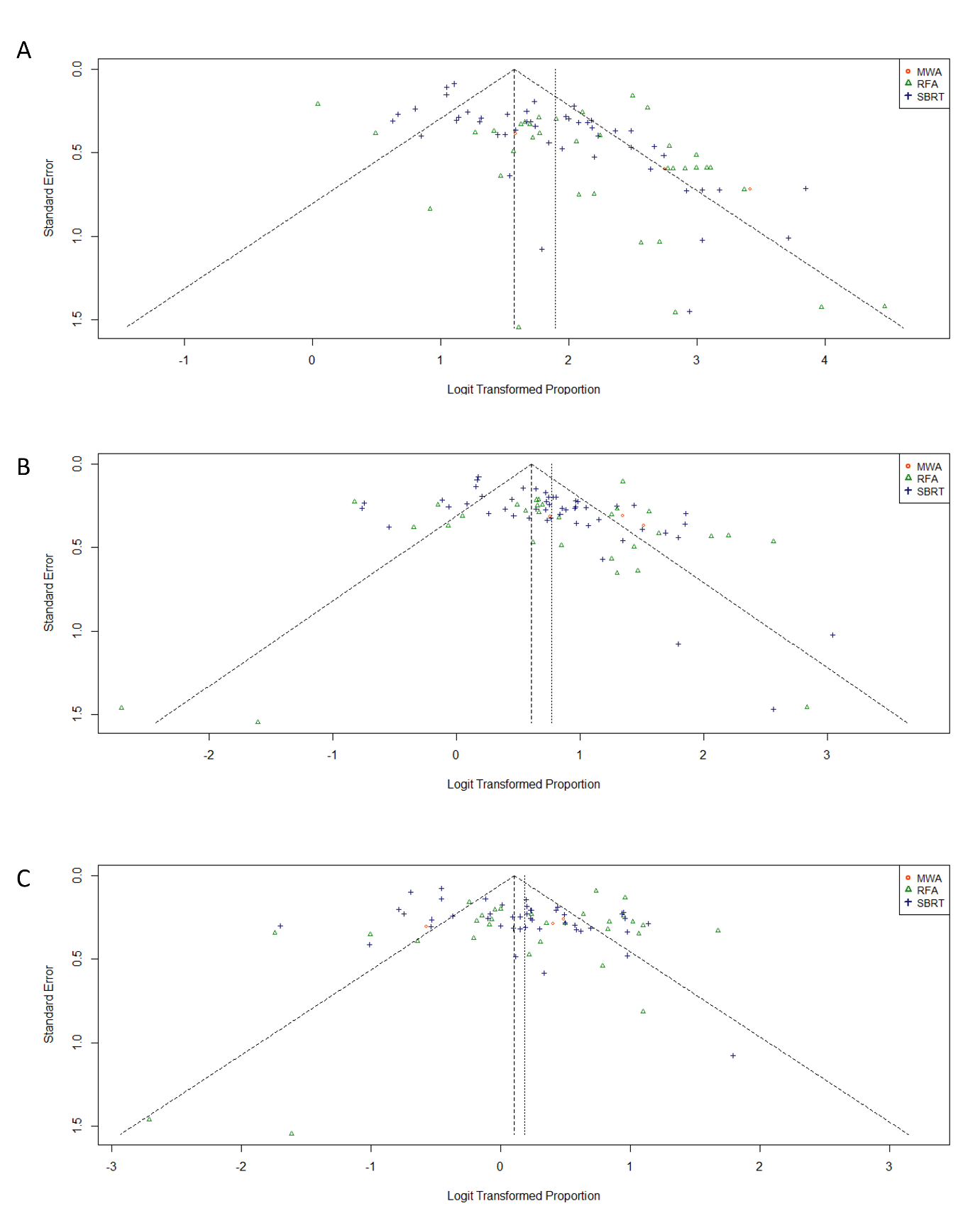


Abbreviations: MWA=microwave ablation; OS=overall survival; RFA=radiofrequency ablation; SBRT=stereotactic body radiation therapy.

# Included Studies

Table 12: List of Included Studies and their MINORS Score

| **First Author** | **Year** | **Title** |
| --- | --- | --- |
| **MWA Studies** | | |
| Kurilova^[1](#_ENREF_1)^ | 2018 | Microwave Ablation in the Management of Colorectal Cancer Pulmonary Metastases |
| Nour-Eldin, N.[^2^](#_ENREF_2) | 2017 | Ablation therapy of non-colorectal cancer lung metastases: retrospective analysis of tumour response post-laser-induced interstitial thermotherapy (LITT), radiofrequency ablation (RFA) and microwave ablation (MWA) |
| Vogl[^3^](#_ENREF_3) | 2018 | A comparison between 915 MHz and 2450 MHz microwave ablation systems for the treatment of small diameter lung metastases |
| Vogl, T.[^4^](#_ENREF_4) | 2016 | Thermal Ablation of Colorectal Lung Metastases: Retrospective Comparison Among Laser-Induced Thermotherapy, Radiofrequency Ablation, and Microwave Ablation |
| Zheng[^5^](#_ENREF_5) | 2016 | Local Efficacy and Survival after Microwave Ablation of Lung Tumors: A Retrospective Study in 183 Patients |
| **RFA Studies** | | |
| Akhan[^6^](#_ENREF_6) | 2016 | Radiofrequency ablation for lung tumors: outcomes, effects on survival, and prognostic factors |
| Ambrogil^[7](#_ENREF_7)^ | 2006 | Percutaneous radiofrequency ablation of lung tumors: results in the mid-term |
| Bonichon^[8](#_ENREF_8)^ | 2013 | Diagnostic accuracy of 18F-FDG PET/CT for assessing response to radiofrequency ablation treatment in lung metastases: a multicentre prospective study |
| Chen[^9^](#_ENREF_9) | 2017 | Clinical assessment of computed tomography guided radiofrequency ablation in the treatment of inoperable patients with pulmonary tumors |
| Chua[^10^](#_ENREF_10) | 2010 | Radiofrequency Ablation as an Adjunct to Systemic Chemotherapy for Colorectal Pulmonary Metastases |
| de Baere T[^11^](#_ENREF_11) | 2015 | Radiofrequency ablation is a valid treatment option for lung metastases: experience in 556 patients with 1037 metastases |
| Fanucchi O[^12^](#_ENREF_12) | 2016 | Long-term results of percutaneous radiofrequency ablation of pulmonary metastases: a single institution experience |
| Ferguson J[^13^](#_ENREF_13) | 2015 | Long term results of RFA to lung metastases from colorectal cancer in 157 patients |
| Gonnet[^14^](#_ENREF_14) | 2019 | Renal cell carcinoma lung metastases treated by radiofrequency ablation integrated with systemic treatments: Over 10 years of experience |
| Hasegawa[^15^](#_ENREF_15) | 2020 | Three-year Survival Rate after Radiofrequency Ablation for Surgically Resectable Colorectal Lung Metastases: A prospective multicenter study |
| Hiyoshi Y[^16^](#_ENREF_16) | 2019 | CT-guided percutaneous radiofrequency ablation for lung metastases from colorectal cancer |
| L. Huang[^17^](#_ENREF_17) | 2011 | Is radiofrequency thermal ablation a safe and effective procedure in the treatment of pulmonary malignancies? |
| Kodama[^18^](#_ENREF_18) | 2015 | Radiofrequency Ablation Using a Multiple-Electrode Switching System for Lung Tumors with 2.0–5.0-cm Maximum Diameter: Phase II Clinical Study |
| Lencioni[^19^](#_ENREF_19) | 2008 | Response to radiofrequency ablation of pulmonary tumours: a prospective, intention-to-treat, multicentre clinical trial (the RAPTURE study) |
| Matsui[^20^](#_ENREF_20) | 2015 | Long-Term Survival following Percutaneous Radiofrequency Ablation of Colorectal Lung Metastases |
| Najafi[^21^](#_ENREF_21) | 2020 | Risk factors for local tumor progression after RFA of pulmonary metastases: a matched case-control study |
| Nour-Eldin, N.[^2^](#_ENREF_2) | 2017 | Ablation therapy of non-colorectal cancer lung metastases: retrospective analysis of tumour response post-laser-induced interstitial thermotherapy (LITT), radiofrequency ablation (RFA) and microwave ablation (MWA) |
| Omae[^22^](#_ENREF_22) | 2016 | Long-Term Survival after Radiofrequency Ablation of Lung Oligometastases from 5 types of Primary Lesions: A Retrospective Evaluation |
| Pennathur[^23^](#_ENREF_23) | 2009 | Image-Guided Radiofrequency Ablation of Lung Neoplasm in 100 Consecutive Patients by a Thoracic Surgical Service |
| Picchi[^24^](#_ENREF_24) | 2020 | RFA of primary and metastatic lung tumors: long-term results |
| Sato[^25^](#_ENREF_25) | 2016 | Radiofrequency ablation of pulmonary metastases from sarcoma: single-center retrospective evaluation of 46 patients |
| Simon[^26^](#_ENREF_26) | 2007 | Pulmonary radiofrequency ablation: Long-term safety and efficacy in 153 patients. |
| Vogl, T.[^4^](#_ENREF_4) | 2016 | Thermal Ablation of Colorectal Lung Metastases: Retrospective Comparison Among Laser-Induced Thermotherapy, Radiofrequency Ablation, and Microwave Ablation |
| Von Meyenfeldt^[27](#_ENREF_27)^ | 2011 | Local Progression After Radiofrequency Ablation for Pulmonary Metastases |
| Wang[^28^](#_ENREF_28) | 2015 | A prospective clinical trial of radiofrequency ablation for pulmonary metastases |
| Yamakado^[29](#_ENREF_29)^ | 2007 | Radiofrequency ablation for the treatment of unresectable lung metastases I npatients with colorectal cancer: a multicenter study in Japan |
| Yamakado^[30](#_ENREF_30)^ | 2009 | Long-term results of radiofrequency ablation in colorectal lung metastases: single center experience |
| Yan[^31^](#_ENREF_31) | 2007 | Treatment Failure After Percutaneous Radiofrequency Ablation for Nonsurgical Candidates With Pulmonary Metastases From Colorectal Carcinoma |
| Zhong[^32^](#_ENREF_32) | 2020 | Long-Term Outcomes in Percutaneous Radiofrequency Ablation for Histologically Proven Colorectal Lung Metastasis |
| **SBRT Studies** | | |
| Agolli[^33^](#_ENREF_33) | 2017 | Lung Metastases Treated With Stereotactic Ablative Radiation Therapy in Oligometastatic Colorectal Cancer Patients: Outcomes and Prognostic Factors After Long-Term Follow-Up |
| Ahmed[^34^](#_ENREF_34) | 2018 | Radiosensitivity of lung metastases by primary histology and implications for stereotactic body radiation therapy using the genomically adjusted radiation dose |
| Aitken[^35^](#_ENREF_35) | 2015 | Initial UK Experience of Stereotactic Body Radiotherapy for Extracranial Oligometastases: Can We Change the Therapeutic Paradigm? |
| Aoki[^36^](#_ENREF_36) | 2016 | Stereotactic body radiotherapy for lung metastases as oligo-recurrence: a single institutional study |
| Bae[^37^](#_ENREF_37) | 2012 | High dose stereotactic body radiotherapy using three fractions for colorectal oligometastases |
| Baumann[^38^](#_ENREF_38) | 2018 | Clinical Results of Mean GTV Dose Optimized Robotic-Guided Stereotactic Body Radiation Therapy for Lung Tumors |
| Benson[^39^](#_ENREF_39) | 2021 | Local Recurrence Outcomes of Colorectal Cancer Oligometastases Treated With Stereotactic Ablative Radiotherapy |
| Berkovic[^40^](#_ENREF_40) | 2020 | Stereotactic robotic body radiotherapy for patients with oligorecurrent pulmonary metastases |
| Binkley[^41^](#_ENREF_41) | 2015 | Colorectal histology is associated with an increased risk of local failure in lung metastases treated with stereotactic ablative radiation therapy. |
| Comito[^42^](#_ENREF_42) | 2014 | Stereotactic Ablative Radiotherapy (SABR) in inoperable oligometastatic disease from colorectal cancer: a safe and effective approach |
| Eriguchi^[43](#_ENREF_43)^ | 2021 | Suitability of Metastatic Lung Tumors for Stereotactic Body Radiotherapy |
| Filippi[^44^](#_ENREF_44) | 2014 | Outcomes of Single Fraction Stereotactic Ablative Radiotherapy for Lung Metastases |
| Filippi[^45^](#_ENREF_45) | 2015 | Stereotactic Ablative Radiation Therapy as First Local Therapy for Lung Oligometastases From Colorectal Cancer: A Single-Institution Cohort Study |
| Franceschini[^46^](#_ENREF_46) | 2017 | Role of stereotactic body radiation therapy for lung metastases from radio-resistant primary tumours |
| Garcia- Cabezas[^47^](#_ENREF_47) | 2015 | Lung metastases in oligometastatic patients:outcome with sterotactic body radiotherapy (SBRT) |
| Geary[^48^](#_ENREF_48) | 2021 | Stereotactic ablative radiotherapy for early stage lung cancer and lung metastases in a New Zealand population |
| Guckenberger[^49^](#_ENREF_49) | 2009 | Dose–Response Relationship for Image-Guided Stereotactic Body Radiotherapy of Pulmonary Tumors: Relevance Of 4D Dose Calculation |
| Helou[^50^](#_ENREF_50) | 2017 | Stereotactic Ablative Radiation Therapy for Pulmonary Metastases: Histology, Dose, and Indication Matter |
| Hof[^51^](#_ENREF_51) | 2007 | Stereotactic Single-Dose Radiotherapy of Lung Metastases |
| Inoue T.[^52^](#_ENREF_52) | 2013 | Stereotactic body radiotherapy for pulmonary metastases Prognostic factors and adverse respiratory events |
| Jang[^53^](#_ENREF_53) | 2017 | Clinical outcomes of stereotactic ablative radiotherapy in patients with pulmonary metastasis |
| Janssen[^54^](#_ENREF_54) | 2016 | Stereotactic Body Radiotherapy Provides Excellent Long-Term Local Control of Very Few Lung Metastases |
| Jingu[^55^](#_ENREF_55) | 2017 | Dose Escalation Improves Outcome in Stereotactic Body Radiotherapy for Pulmonary Oligometastases from Colorectal Cancer |
| Jung[^56^](#_ENREF_56) | 2015 | Clinical efficacy of stereotactic ablative radiotherapy for lung metastases arising from colorectal cancer |
| Kessel[^57^](#_ENREF_57) | 2020 | Stereotactic body radiotherapy (SBRT) in patients with lung metastases – prognostic factors and long-term survival using patient self-reported outcome (PRO) |
| Kinj^[58](#_ENREF_58)^ | 2017 | Radiosensitivity of Colon and Rectal Lung Oligometastasis Treated With Stereotactic Ablative Radiotherapy |
| Korzets Ceder[^59^](#_ENREF_59) | 2018 | Stereotactic body radiotherapy for central lung tumors, yes we can! |
| Lancia[^60^](#_ENREF_60) | 2019 | Oligometastatic cancer in elderly patients: the “blitzkrieg” radiotherapy approach |
| Lindsay[^61^](#_ENREF_61) | 2018 | Treatment of Sarcoma Lung Metastases with Stereotactic Body Radiotherapy |
| Lodeweges^[62](#_ENREF_62)^ | 2017 | Long-term Outcome of Surgery or Stereotactic Radiotherapy for Lung Oligometastases |
| Loi[^63^](#_ENREF_63) | 2021 | Dose coverage impacts local control in ultra-central lung oligometastases treated with stereotactic radiotherapy |
| Mazzola[^64^](#_ENREF_64) | 2018 | Stereotactic body radiotherapy for lung oligometastases impacts on systemic treatment-free survival: a cohort study |
| Mazzola[^65^](#_ENREF_65) | 2019 | Stereotactic body radiotherapy of central lung malignancies using a simultaneous integrated protection approach |
| Navarria^[66](#_ENREF_66)^ | 2014 | Stereotactic body radiotherapy (sbrt) in lung oligometastatic patients: role of local treatments |
| Nicosia[^67^](#_ENREF_67) | 2021 | A multicenter LArge retrospectIve daTabase on the personalization of stereotactic ABlative radiotherapy use in lung metastases from colon-rectal cancer: The LaIT-SABR study |
| Nuyttens^[68](#_ENREF_68)^ | 2012 | Outcome of four-dimensional stereotactic radiotherapy for centrally located lung tumors |
| Oh[^69^](#_ENREF_69) | 2012 | Potentially curative stereotactic body radiation therapy (SBRT) for single or oligometastasis to the lung |
| Okunieff^[70](#_ENREF_70)^ | 2006 | Stereotactic Body Radiation Therapy (SBRT) for lung metastases |
| Osti[^71^](#_ENREF_71) | 2013 | Clinical outcomes of single dose stereotactic radiotherapy for lung metastases |
| Osti[^72^](#_ENREF_72) | 2018 | 30 Gy single dose stereotactic body radiation therapy (SBRT): Report on outcome in a large series of patients with lung oligometastatic disease |
| Parker[^73^](#_ENREF_73) | 2019 | Impact of Tumor Size on Local Control and Pneumonitis After Stereotactic Body Radiation Therapy for Lung Tumors |
| Ricardi[^74^](#_ENREF_74) | 2012 | Stereotactic body radiotherapy for lung metastases |
| Ricco[^75^](#_ENREF_75) | 2017 | Lung metastases treated with stereotactic body radiotherapy: the RSSearch® patient Registry’s experience |
| Rieber[^76^](#_ENREF_76) | 2016 | Stereotactic body radiotherapy (SBRT) for medically inoperable lung metastases-A pooled analysis of the German working group "stereotactic radiotherapy" |
| Scorsetti^[77](#_ENREF_77)^ | 2015 | The role of stereotactic body radiation therapy (SBRT) in the treatment of oligometastatic disease in the elderly |
| Sharma[^78^](#_ENREF_78) | 2018 | Factors affecting local control of pulmonary oligometastases treated with stereotactic body radiotherapy |
| Sharma[^79^](#_ENREF_79) | 2019 | Survival and prognostic factors of pulmonary oligometastases treated with stereotactic body radiotherapy |
| Siva[^80^](#_ENREF_80) | 2015 | Comparison of Single-fraction and Multi-fraction Stereotactic Radiotherapy for Patients with 18F-fluorodeoxyglucose Positron Emission Tomography-staged Pulmonary Oligometastases |
| Siva[^81^](#_ENREF_81) | 2021 | Single-Fraction vs Multifraction Stereotactic Ablative Body Radiotherapy for Pulmonary Oligometastases (SAFRON II) |
| Takahashi[^82^](#_ENREF_82) | 2012 | Stereotactic body radiotherapy for metastatic lung cancer as oligo-recurrence: an analysis of 42 cases |
| Takeda[^83^](#_ENREF_83) | 2011 | Stereotactic body radiotherapy (SBRT) for oligometastatic lung tumors from colorectal cancer and other primary cancers in comparison with primary lung cancer |
| Tekatli^[84](#_ENREF_84)^ | 2017 | Optimizing SABR delivery for synchronous multiple lung tumors using volumetric-modulated arc therapy |
| Wang[^85^](#_ENREF_85) | 2015 | Clinical outcomes of cyberknife stereotactic radiosurgery for lung metastases |
| Yamamoto[^86^](#_ENREF_86) | 2014 | Outcomes after stereotactic body radiotherapy for lung tumors, with emphasis on comparison of primary lung cancer and metastatic lung tumors |
| Yamashita[^87^](#_ENREF_87) | 2016 | Lung stereotactic radiotherapy for oligometastases: comparison of oligo-recurrence and sync-oligometastases |
| Zhang[^88^](#_ENREF_88) | 2011 | Stereotactic body radiation therapy favors long-term overall survival in patients with lung metastases: five-year experience of a single-institution |

Reference List

^1^Kurilova IG-A, A. Beets-Tan, R. G. Erinjeri, J. Petre, E. N. Gonen, M. Bains, M. Kemeny, N. E. Solomon, S. B. Sofocleous, C. T. (2018) Microwave Ablation in the Management of Colorectal Cancer Pulmonary Metastases. *CardioVascular and Interventional Radiology* 41 (10): 1530-1544.

^2^Nour-Eldin N-EA, Exner S, Al-Subhi M, Naguib NN, Kaltenbach B et al. (2017) Ablation therapy of non-colorectal cancer lung metastases: retrospective analysis of tumour response post-laser-induced interstitial thermotherapy (LITT), radiofrequency ablation (RFA) and microwave ablation (MWA). *International Journal of Hyperthermia* 33 (7): 820-829.

^3^Vogl TJR, A. Nour-Eldin, N. E. A. Hohenforst-Schmidt, W. Bednarova, I. Kaltenbach, B. (2018) A comparison between 915 MHz and 2450 MHz microwave ablation systems for the treatment of small diameter lung metastases. *Diagnostic and Interventional Radiology* 24 (1): 31-37.

^4^Vogl TJ, Eckert R, Naguib NN, Beeres M, Gruber-Rouh T et al. (2016) Thermal Ablation of Colorectal Lung Metastases: Retrospective Comparison Among Laser-Induced Thermotherapy, Radiofrequency Ablation, and Microwave Ablation. *AJR Am J Roentgenol* 207 (6): 1340-1349.

^5^Zheng A, Ye X, Yang X, Huang G, Gai Y (2016) Local Efficacy and Survival after Microwave Ablation of Lung Tumors: A Retrospective Study in 183 Patients. *J Vasc Interv Radiol* 27 (12): 1806-1814.

^6^Akhan O, Güler E, Akıncı D, Çiftçi T, Köse I (2016) Radiofrequency ablation for lung tumors: outcomes, effects on survival, and prognostic factors. *Diagn Interv Radiol* 22 (1): 65-71.

^7^Ambrogi MC, Lucchi M, Dini P, Melfi F, Fontanini G et al. (2006) Percutaneous radiofrequency ablation of lung tumours: results in the mid-term. *Eur J Cardiothorac Surg* 30 (1): 177-183.

^8^Bonichon F, Palussiere J, Godbert Y, Pulido M, Descat E et al. (2013) Diagnostic accuracy of 18F-FDG PET/CT for assessing response to radiofrequency ablation treatment in lung metastases: a multicentre prospective study. *Eur J Nucl Med Mol Imaging* 40 (12): 1817-1827.

^9^Chen T, Jin J, Chen S (2017) Clinical assessment of computed tomography guided radiofrequency ablation in the treatment of inoperable patients with pulmonary tumors. *J Thorac Dis* 9 (12): 5131-5142.

^10^Chua TC, Thornbury K, Saxena A, Liauw W, Glenn D et al. (2010) Radiofrequency ablation as an adjunct to systemic chemotherapy for colorectal pulmonary metastases. *Cancer* 116 (9): 2106-2114.

^11^de Baère T, Aupérin A, Deschamps F, Chevallier P, Gaubert Y et al. (2015) Radiofrequency ablation is a valid treatment option for lung metastases: experience in 566 patients with 1037 metastases. *Ann Oncol* 26 (5): 987-991.

^12^Fanucchi O, Ambrogi MC, Aprile V, Cioni R, Cappelli C et al. (2016) Long-term results of percutaneous radiofrequency ablation of pulmonary metastases: a single institution experience. *Interact Cardiovasc Thorac Surg* 23 (1): 57-64.

^13^Ferguson J, Alzahrani N, Zhao J, Glenn D, Power M et al. (2015) Long term results of RFA to lung metastases from colorectal cancer in 157 patients. *Eur J Surg Oncol* 41 (5): 690-695.

^14^Gonnet A, Salabert L, Roubaud G, Catena V, Brouste V et al. (2019) Renal cell carcinoma lung metastases treated by radiofrequency ablation integrated with systemic treatments: Over 10 years of experience. *BMC Cancer* 19 (1):

^15^Hasegawa TT, H. Kodama, H. Yamanaka, T. Nakatsuka, A. Sato, Y. Takao, M. Katayama, Y. Fukai, I. Kato, T. Tokui, T. Tempaku, H. Adachi, K. Matsushima, Y. Inaba, Y. Yamakado, K. (2020) Three-year Survival Rate after Radiofrequency Ablation for Surgically Resectable Colorectal Lung Metastases: A prospective multicenter study. *Radiology* 294 (2): 686-695.

^16^Hiyoshi Y, Miyamoto Y, Kiyozumi Y, Sawayama H, Eto K et al. (2019) CT-guided percutaneous radiofrequency ablation for lung metastases from colorectal cancer. *Int J Clin Oncol* 24 (3): 288-295.

^17^Huang L, Han Y, Zhao J, Wang X, Cheng Q et al. (2011) Is radiofrequency thermal ablation a safe and effective procedure in the treatment of pulmonary malignancies? *Eur J Cardiothorac Surg* 39 (3): 348-351.

^18^Kodama H, Yamakado K, Hasegawa T, Fujimori M, Yamanaka T et al. (2015) Radiofrequency Ablation Using a Multiple-Electrode Switching System for Lung Tumors with 2.0-5.0-cm Maximum Diameter: Phase II Clinical Study. *Radiology* 277 (3): 895-902.

^19^Lencioni R, Crocetti L, Cioni R, Suh R, Glenn D et al. (2008) Response to radiofrequency ablation of pulmonary tumours: a prospective, intention-to-treat, multicentre clinical trial (the RAPTURE study). *Lancet Oncol* 9 (7): 621-628.

^20^Matsui Y, Hiraki T, Gobara H, Iguchi T, Fujiwara H et al. (2015) Long-term survival following percutaneous radiofrequency ablation of colorectal lung metastases. *J Vasc Interv Radiol* 26 (3): 303-310;quiz 311.

^21^Najafi A, de Baere T, Purenne E, Bayar A, Al Ahmar M et al. (2021) Risk factors for local tumor progression after RFA of pulmonary metastases: a matched case-control study. *Eur Radiol*

^22^Omae K, Hiraki T, Gobara H, Iguchi T, Fujiwara H et al. (2016) Long-Term Survival after Radiofrequency Ablation of Lung Oligometastases from Five Types of Primary Lesions: A Retrospective Evaluation. *J Vasc Interv Radiol* 27 (9): 1362-1370.

^23^Pennathur A, Abbas G, Gooding WE, Schuchert MJ, Gilbert S et al. (2009) Image-guided radiofrequency ablation of lung neoplasm in 100 consecutive patients by a thoracic surgical service. *Ann Thorac Surg* 88 (5): 1601-1606; discussion 1607-1608.

^24^Picchi SGL, G. Bianco, A. Coppola, A. Ierardi, A. M. Rossi, U. G. Lassandro, F. (2020) RFA of primary and metastatic lung tumors: long-term results. *Medical Oncology* 37 (5):

^25^Sato T, Iguchi T, Hiraki T, Gobara H, Fujiwara H et al. (2017) Radiofrequency ablation of pulmonary metastases from sarcoma: single-center retrospective evaluation of 46 patients. *Jpn J Radiol* 35 (2): 61-67.

^26^Simon CJ, Dupuy DE, DiPetrillo TA, Safran HP, Grieco CA et al. (2007) Pulmonary radiofrequency ablation: long-term safety and efficacy in 153 patients. *Radiology* 243 (1): 268-275.

^27^von Meyenfeldt EM, Prevoo W, Peyrot D, Lai AFN, Burgers SJ et al. (2011) Local progression after radiofrequency ablation for pulmonary metastases. *Cancer* 117 (16): 3781-3787.

^28^Wang Y, Lu X, Wang Y, Li W, Li G et al. (2015) A prospective clinical trial of radiofrequency ablation for pulmonary metastases. *Mol Clin Oncol* 3 (3): 559-562.

^29^Yamakado K, Hase S, Matsuoka T, Tanigawa N, Nakatsuka A et al. (2007) Radiofrequency ablation for the treatment of unresectable lung metastases in patients with colorectal cancer: a multicenter study in Japan. *J Vasc Interv Radiol* 18 (3): 393-398.

^30^Yamakado K, Inoue Y, Takao M, Takaki H, Nakatsuka A et al. (2009) Long-term results of radiofrequency ablation in colorectal lung metastases: single center experience. *Oncol Rep* 22 (4): 885-891.

^31^Yan TD, King J, Sjarif A, Glenn D, Steinke K et al. (2007) Treatment failure after percutaneous radiofrequency ablation for nonsurgical candidates with pulmonary metastases from colorectal carcinoma. *Ann Surg Oncol* 14 (5): 1718-1726.

^32^Zhong J, Palkhi E, Ng H, Wang K, Milton R et al. (2020) Long-Term Outcomes in Percutaneous Radiofrequency Ablation for Histologically Proven Colorectal Lung Metastasis. *Cardiovasc Intervent Radiol* 43 (12): 1900-1907.

^33^Agolli L, Bracci S, Nicosia L, Valeriani M, De Sanctis V et al. (2017) Lung Metastases Treated With Stereotactic Ablative Radiation Therapy in Oligometastatic Colorectal Cancer Patients: Outcomes and Prognostic Factors After Long-Term Follow-Up. *Clin Colorectal Cancer* 16 (1): 58-64.

^34^Ahmed KA, Scott JG, Arrington JA, Naghavi AO, Grass GD et al. (2018) Radiosensitivity of Lung Metastases by Primary Histology and Implications for Stereotactic Body Radiation Therapy Using the Genomically Adjusted Radiation Dose. *J Thorac Oncol* 13 (8): 1121-1127.

^35^Aitken K, Tree A, Thomas K, Nutting C, Hawkins M et al. (2015) Initial UK Experience of Stereotactic Body Radiotherapy for Extracranial Oligometastases: Can We Change the Therapeutic Paradigm? *Clin Oncol (R Coll Radiol)* 27 (7): 411-419.

^36^Aoki M, Hatayama Y, Kawaguchi H, Hirose K, Sato M et al. (2016) Stereotactic body radiotherapy for lung metastases as oligo-recurrence: a single institutional study. *J Radiat Res* 57 (1): 55-61.

^37^Bae SH, Kim MS, Cho CK, Kang JK, Kang HJ et al. (2012) High dose stereotactic body radiotherapy using three fractions for colorectal oligometastases. *J Surg Oncol* 106 (2): 138-143.

^38^Baumann R, Chan MKH, Pyschny F, Stera S, Malzkuhn B et al. (2018) Clinical Results of Mean GTV Dose Optimized Robotic-Guided Stereotactic Body Radiation Therapy for Lung Tumors. *Front Oncol* 8 171.

^39^Benson KRK, Sandhu N, Zhang C, Ko R, Toesca DAS et al. (2021) Local Recurrence Outcomes of Colorectal Cancer Oligometastases Treated With Stereotactic Ablative Radiotherapy. *Am J Clin Oncol* 44 (11): 559-564.

^40^Berkovic P, Gulyban A, Defraene G, Swenen L, Dechambre D et al. (2020) Stereotactic robotic body radiotherapy for patients with oligorecurrent pulmonary metastases. *BMC Cancer* 20 (1): 402.

^41^Binkley MS, Trakul N, Jacobs LR, von Eyben R, Le QT et al. (2015) Colorectal Histology Is Associated With an Increased Risk of Local Failure in Lung Metastases Treated With Stereotactic Ablative Radiation Therapy. *Int J Radiat Oncol Biol Phys* 92 (5): 1044-1052.

^42^Comito T, Cozzi L, Clerici E, Campisi MC, Liardo RL et al. (2014) Stereotactic Ablative Radiotherapy (SABR) in inoperable oligometastatic disease from colorectal cancer: a safe and effective approach. *BMC Cancer* 14 619.

^43^Eriguchi T, Tsukamoto N, Kumabe A, Ogata T, Inoue Y et al. (2022) Suitability of Metastatic Lung Tumors for Stereotactic Body Radiotherapy. *Cancer Invest* 40 (4): 378-386.

^44^Filippi AR, Badellino S, Guarneri A, Levis M, Botticella A et al. (2014) Outcomes of single fraction stereotactic ablative radiotherapy for lung metastases. *Technol Cancer Res Treat* 13 (1): 37-45.

^45^Filippi AR, Badellino S, Ceccarelli M, Guarneri A, Franco P et al. (2015) Stereotactic ablative radiation therapy as first local therapy for lung oligometastases from colorectal cancer: a single-institution cohort study. *Int J Radiat Oncol Biol Phys* 91 (3): 524-529.

^46^Franceschini D, Cozzi L, De Rose F, Navarria P, Franzese C et al. (2017) Role of stereotactic body radiation therapy for lung metastases from radio-resistant primary tumours. *J Cancer Res Clin Oncol* 143 (7): 1293-1299.

^47^Garcia-Cabezas S, Bueno C, Rivin E, Roldan JM, Palacios-Eito A (2015) Lung metastases in oligometastatic patients: outcome with stereotactic body radiation therapy (SBRT). *Clin Transl Oncol* 17 (8): 668-672.

^48^Geary RL, Yasin N, Lin F, Whalley D, Thotathil Z et al. (2021) Stereotactic ablative radiotherapy for early stage lung cancer and lung metastases in a New Zealand population. *New Zealand Medical Journal* 134 (1529): 45-56.

^49^Guckenberger M, Wulf J, Mueller G, Krieger T, Baier K et al. (2009) Dose-response relationship for image-guided stereotactic body radiotherapy of pulmonary tumors: relevance of 4D dose calculation. *Int J Radiat Oncol Biol Phys* 74 (1): 47-54.

^50^Helou J, Thibault I, Poon I, Chiang A, Jain S et al. (2017) Stereotactic Ablative Radiation Therapy for Pulmonary Metastases: Histology, Dose, and Indication Matter. *Int J Radiat Oncol Biol Phys* 98 (2): 419-427.

^51^Hof H, Hoess A, Oetzel D, Debus J, Herfarth K (2007) Stereotactic single-dose radiotherapy of lung metastases. *Strahlenther Onkol* 183 (12): 673-678.

^52^Inoue T, Oh RJ, Shiomi H, Masai N, Miura H (2013) Stereotactic body radiotherapy for pulmonary metastases. Prognostic factors and adverse respiratory events. *Strahlenther Onkol* 189 (4): 285-292.

^53^Jang BS, Kim HJ, Kim BH, Kim DW, Kim YT et al. (2017) Clinical outcomes of stereotactic ablative radiotherapy in patients with pulmonary metastasis. *Jpn J Clin Oncol* 47 (1): 61-66.

^54^Janssen S, Kasmann L, Rudat V, Rades D (2016) Stereotactic Body Radiotherapy Provides Excellent Long-Term Local Control of Very Few Lung Metastases. *In Vivo* 30 (2): 155-157.

^55^Jingu K, Matsuo Y, Onishi H, Yamamoto T, Aoki M et al. (2017) Dose Escalation Improves Outcome in Stereotactic Body Radiotherapy for Pulmonary Oligometastases from Colorectal Cancer. *Anticancer Res* 37 (5): 2709-2713.

^56^Jung J, Song SY, Kim JH, Yu CS, Kim JC et al. (2015) Clinical efficacy of stereotactic ablative radiotherapy for lung metastases arising from colorectal cancer. *Radiat Oncol* 10 238.

^57^Kessel KAG, R. C. E. Kraus, K. M. Hoffmann, H. Oechsner, M. Combs, S. E. (2020) Stereotactic body radiotherapy (SBRT) in patients with lung metastases - prognostic factors and long-term survival using patient self-reported outcome (PRO). *BMC Cancer* 20 (1): 442.

^58^Kinj R, Bondiau PY, François E, Gérard JP, Naghavi AO et al. (2017) Radiosensitivity of Colon and Rectal Lung Oligometastasis Treated With Stereotactic Ablative Radiotherapy. *Clin Colorectal Cancer* 16 (3): e211-e220.

^59^Korzets Ceder Y, Fenig E, Popvtzer A, Peled N, Kramer MR et al. (2018) Stereotactic body radiotherapy for central lung tumors, yes we can! *Radiat Oncol* 13 (1): 77.

^60^Lancia A, Ingrosso G, Carosi A, Bottero M, Cancelli A et al. (2019) Oligometastatic cancer in elderly patients: the "blitzkrieg" radiotherapy approach : SBRT in oligometastatic elderly patients. *Aging Clin Exp Res* 31 (1): 109-114.

^61^Lindsay AD, Haupt EE, Chan CM, Spiguel AR, Scarborough MT et al. (2018) Treatment of Sarcoma Lung Metastases with Stereotactic Body Radiotherapy. *Sarcoma* 2018 9132359.

^62^Lodeweges JE, Klinkenberg TJ, Ubbels JF, Groen HJM, Langendijk JA et al. (2017) Long-term Outcome of Surgery or Stereotactic Radiotherapy for Lung Oligometastases. *J Thorac Oncol* 12 (9): 1442-1445.

^63^Loi M, Franceschini D, Dominici L, Chiola I, Franzese C et al. (2020) Dose coverage impacts local control in ultra-central lung oligometastases treated with stereotactic radiotherapy. *Strahlentherapie und Onkologie* 24 24.

^64^Mazzola R, Fersino S, Ferrera G, Targher G, Figlia V et al. (2018) Stereotactic body radiotherapy for lung oligometastases impacts on systemic treatment-free survival: a cohort study. *Med Oncol* 35 (9): 121.

^65^Mazzola R, Ruggieri R, Figlia V, Rigo M, Giaj Levra N et al. (2019) Stereotactic body radiotherapy of central lung malignancies using a simultaneous integrated protection approach : A prospective observational study. *Strahlenther Onkol* 195 (8): 719-724.

^66^Navarria P, Ascolese AM, Tomatis S, Cozzi L, De Rose F et al. (2014) Stereotactic body radiotherapy (sbrt) in lung oligometastatic patients: role of local treatments. *Radiat Oncol* 9 (1): 91.

^67^Nicosia L, Franceschini D, Perrone-Congedi F, Casamassima F, Gerardi MA et al. (2022) A multicenter LArge retrospectIve daTabase on the personalization of stereotactic ABlative radiotherapy use in lung metastases from colon-rectal cancer: The LaIT-SABR study. *Radiother Oncol* 166 92-99.

^68^Nuyttens JJ, van der Voort van Zyp NC, Praag J, Aluwini S, van Klaveren RJ et al. (2012) Outcome of four-dimensional stereotactic radiotherapy for centrally located lung tumors. *Radiother Oncol* 102 (3): 383-387.

^69^Oh D, Ahn YC, Seo JM, Shin EH, Park HC et al. (2012) Potentially curative stereotactic body radiation therapy (SBRT) for single or oligometastasis to the lung. *Acta Oncol* 51 (5): 596-602.

^70^Okunieff P, Petersen AL, Philip A, Milano MT, Katz AW et al. (2006) Stereotactic Body Radiation Therapy (SBRT) for lung metastases. *Acta Oncol* 45 (7): 808-817.

^71^Osti MF, Carnevale A, Valeriani M, De Sanctis V, Minniti G et al. (2013) Clinical outcomes of single dose stereotactic radiotherapy for lung metastases. *Clin Lung Cancer* 14 (6): 699-703.

^72^Osti MF, Agolli L, Valeriani M, Reverberi C, Bracci S et al. (2018) 30 Gy single dose stereotactic body radiation therapy (SBRT): Report on outcome in a large series of patients with lung oligometastatic disease. *Lung Cancer* 122 165-170.

^73^Parker SM, Siochi RA, Wen S, Mattes MD (2019) Impact of Tumor Size on Local Control and Pneumonitis After Stereotactic Body Radiation Therapy for Lung Tumors. *Pract Radiat Oncol* 9 (1): e90-e97.

^74^Ricardi U, Filippi AR, Guarneri A, Ragona R, Mantovani C et al. (2012) Stereotactic body radiation therapy for lung metastases. *Lung Cancer* 75 (1): 77-81.

^75^Ricco A, Davis J, Rate W, Yang J, Perry D et al. (2017) Lung metastases treated with stereotactic body radiotherapy: the RSSearch® patient Registry's experience. *Radiat Oncol* 12 (1): 35.

^76^Rieber J, Streblow J, Uhlmann L, Flentje M, Duma M et al. (2016) Stereotactic body radiotherapy (SBRT) for medically inoperable lung metastases-A pooled analysis of the German working group "stereotactic radiotherapy". *Lung Cancer* 97 51-58.

^77^Scorsetti M, Clerici E, Navarria P, D'Agostino G, Piergallini L et al. (2015) The role of stereotactic body radiation therapy (SBRT) in the treatment of oligometastatic disease in the elderly. *Br J Radiol* 88 (1053): 20150111.

^78^Sharma A, Duijm M, Oomen-de Hoop E, Aerts JG, Verhoef C et al. (2018) Factors affecting local control of pulmonary oligometastases treated with stereotactic body radiotherapy. *Acta Oncol* 57 (8): 1031-1037.

^79^Sharma A, Duijm M, Oomen-de Hoop E, Aerts JG, Verhoef C et al. (2019) Survival and prognostic factors of pulmonary oligometastases treated with stereotactic body radiotherapy. *Acta Oncol* 58 (1): 74-80.

^80^Siva S, Kirby K, Caine H, Pham D, Kron T et al. (2015) Comparison of Single-fraction and Multi-fraction Stereotactic Radiotherapy for Patients with 18F-fluorodeoxyglucose Positron Emission Tomography-staged Pulmonary Oligometastases. *Clin Oncol (R Coll Radiol)* 27 (6): 353-361.

^81^Siva S, Bressel M, Mai T, Le H, Vinod S et al. (2021) Single-Fraction vs Multifraction Stereotactic Ablative Body Radiotherapy for Pulmonary Oligometastases (SAFRON II): The Trans Tasman Radiation Oncology Group 13.01 Phase 2 Randomized Clinical Trial. *JAMA Oncol* 7 (10): 1476-1485.

^82^Takahashi W, Yamashita H, Niibe Y, Shiraishi K, Hayakawa K et al. (2012) Stereotactic body radiotherapy for metastatic lung cancer as oligo-recurrence: an analysis of 42 cases. *Pulm Med* 2012 454107.

^83^Takeda A, Kunieda E, Ohashi T, Aoki Y, Koike N et al. (2011) Stereotactic body radiotherapy (SBRT) for oligometastatic lung tumors from colorectal cancer and other primary cancers in comparison with primary lung cancer. *Radiother Oncol* 101 (2): 255-259.

^84^Tekatli H, Tetar SU, Nguyen TK, Warner A, Verbakel WF et al. (2017) Optimizing SABR delivery for synchronous multiple lung tumors using volumetric-modulated arc therapy. *Acta Oncol* 56 (4): 548-554.

^85^Wang Z, Kong QT, Li J, Wu XH, Li B et al. (2015) Clinical outcomes of cyberknife stereotactic radiosurgery for lung metastases. *J Thorac Dis* 7 (3): 407-412.

^86^Yamamoto T, Jingu K, Shirata Y, Koto M, Matsushita H et al. (2014) Outcomes after stereotactic body radiotherapy for lung tumors, with emphasis on comparison of primary lung cancer and metastatic lung tumors. *BMC Cancer* 14 464.

^87^Yamashita H, Niibe Y, Yamamoto T, Katsui K, Jingu K et al. (2016) Lung stereotactic radiotherapy for oligometastases: comparison of oligo-recurrence and sync-oligometastases. *Jpn J Clin Oncol* 46 (7): 687-691.

^88^Zhang Y, Xiao JP, Zhang HZ, Yin WB, Hu YM et al. (2011) Stereotactic body radiation therapy favors long-term overall survival in patients with lung metastases: five-year experience of a single-institution. *Chin Med J (Engl)* 124 (24): 4132-4137.
